# Addressing treatment switching in the ALTA-1L trial with g-methods: exploring the impact of model specification

**DOI:** 10.1186/s12874-024-02437-6

**Published:** 2024-12-20

**Authors:** Amani Al Tawil, Sean McGrath, Robin Ristl, Ulrich Mansmann

**Affiliations:** 1 Institute for Medical Information Processing, Biometry, and Epidemiology (IBE), Faculty of Medicine, Ludwig-Maximilians-Universität München, Marchioninistr. 15, 81377 Munich, Germany; 2 Pettenkofer School of Public Health, Faculty of Medicine, Ludwig-Maximilians-Universität München, Elisabeth-Winterhalter-Weg 6, 81377 Munich, Germany; 3 Department of Biostatistics, Harvard T.H. Chan School of Public Health, 677 Huntington Ave, MA 02115 Boston, USA; 4 Center for Medical Data Science, Medical University of Vienna, Spitalgasse 23, A-1090 Vienna, Austria

**Keywords:** Survival analysis, Treatment switching, Time-dependent confounding, Treatment-confounder feedback, IPCW, Parametric g-formula

## Abstract

**Background:**

Treatment switching in randomized clinical trials introduces challenges in performing causal inference. Intention To Treat (ITT) analyses often fail to fully capture the causal effect of treatment in the presence of treatment switching. Consequently, decision makers may instead be interested in causal effects of hypothetical treatment strategies that do not allow for treatment switching. For example, the phase 3 ALTA-1L trial showed that brigatinib may have improved Overall Survival (OS) compared to crizotinib if treatment switching had not occurred. Their sensitivity analysis using Inverse Probability of Censoring Weights (IPCW), reported a Hazard Ratio (HR) of 0.50 (95% CI, 0.28-0.87), while their initial ITT analysis estimated an HR of 0.81 (0.53-1.22).

**Methods:**

We used a directed acyclic graph to depict the clinical setting of the ALTA-1L trial in the presence of treatment switching, illustrating the concept of treatment-confounder feedback and highlighting the need for g-methods. In a re-analysis of the ALTA-1L trial data, we used IPCW and the parametric g-formula to adjust for baseline and time-varying covariates to estimate the effect of two hypothetical treatment strategies on OS: “always treat with brigatinib” versus “always treat with crizotinib”. We conducted various sensitivity analyses using different model specifications and weight truncation approaches.

**Results:**

Applying the IPCW approach in a series of sensitivity analyses yielded Cumulative HRs (cHRs) ranging between 0.38 (0.12, 0.98) and 0.73 (0.45,1.22) and Risk Ratios (RRs) ranging between 0.52 (0.32, 0.98) and 0.79 (0.54,1.17). Applying the parametric g-formula resulted in cHRs ranging between 0.61 (0.38,0.91) and 0.72 (0.43,1.07) and RRs ranging between 0.71 (0.48,0.94) and 0.79 (0.54,1.05).

**Conclusion:**

Our results consistently indicated that our estimated ITT effect estimate (cHR: 0.82 (0.51,1.22) may have underestimated brigatinib’s benefit by around 10-45 percentage points (using IPCW) and 10-20 percentage points (using the parametric g-formula) across a wide range of model choices. Our analyses underscore the importance of performing sensitivity analyses, as the result from a single analysis could potentially stand as an outlier in a whole range of sensitivity analyses.

**Trial registration:**

Clinicaltrials.gov Identifier: NCT02737501 on April 14, 2016.

**Supplementary Information:**

The online version contains supplementary material available at 10.1186/s12874-024-02437-6.

## Background

### Treatment switching

Despite common misconception, Randomized Controlled Trials (RCTs) are still susceptible to confounding and selection bias [1]. Although randomization protects against baseline confounding, RCTs can still experience post-randomization confounding and selection bias resulting from various factors such as non-compliance with protocols, differential use of concomitant therapies, treatment switching or differential Loss To Follow-Up (LTFU) [1, 2]. In oncology, long-term RCTs aim to investigate the effect of sustained clinical interventions in typical care settings. This brings along a greater risk of post-randomization confounding and attrition bias, potentially compromising the validity of study findings.

Treatment switching typically happens in oncology trials where control group patients are often permitted to crossover to the experimental treatment after disease progression [3–5] when initial analysis indicates that clinical equipoise is no longer maintained. Consequently, clinical trials typically conduct an Intention To Treat (ITT) analysis as their primary analysis. This approach does not necessitate adjusting for post-randomization factors (in the absence of informative LTFU) because it aims to estimate the effect of treatment assigned at baseline. However, when the experimental treatment is superior to the control, the potential benefits received by those who switched to the experimental treatment are not properly accounted for, typically resulting in an underestimation of the treatment effect [6, 7].

### Per-protocol effects and g-methods

Decision makers may instead be interested in the causal effect of the treatment on the outcome had all individuals adhered to their assigned treatment. Estimating causal effects of sustained treatment interventions in the presence of treatment switching and differential LTFU typically requires accounting for time-dependent confounding [8]. Despite their widespread use, standard regression methods that aim to adjust for time-dependent confounding, such as time-dependent Cox Proportional Hazards (CoxPH) models, Generalized Estimating Equations (GEEs) or random-effects models, are typically inadequate for estimating causal effects when time-varying confounders are affected by prior treatment [9]: a phenomenon known as treatment-confounder feedback.

G-methods are a family of methods to estimate causal effects in the presence of treatment-confounder feedback [10]. G-methods include the g-formula [11], Inverse Probability Weighting (IPW) [12], and g-estimation [13]. As such, g-methods may provide more accurate estimates of survival differences had control group subjects not switched treatment and all participants remained under follow-up.

### Motivating example: ALTA-1L trial

#### Study design

The ALK (anaplastic lymphoma kinase) in Lung Cancer Trial of Brigatinib in 1st Line (ALTA-1L) [14] was a randomized, open-label, controlled, phase 3 study evaluating the efficacy of brigatinib (experimental arm) versus crizotinib (control arm) on Progression-Free Survival (PFS). The study population consisted of ALK-positive Non-Small Cell Lung Cancer (NSCLC) patients who had not received prior treatment with an ALK inhibitor. Detailed descriptions of the study design have been previously reported in Camidge 2018 [14].

#### Reported study results

A total of 275 patients were enrolled and randomized to receive either brigatinib ($n=137$) or crizotinib ($n=138$). The primary endpoint was PFS, as assessed by the Blinded Independent Review Committee (BIRC), per Response Evaluation Criteria in Solid Tumors (RECIST v1.1). The ITT results from the ALTA-1L trial based on a stratified CoxPH regression analysis demonstrated a statistically significant delay in PFS for patients in the brigatinib arm (Hazard Ratio (HR)= 0.48 (95% Confidence Interval (CI), 0.35–0.66)). However, ALTA-1L has failed to show a significant difference in Overall Survival (OS) – a secondary endpoint – between the two study arms (HR= 0.81 (0.53–1.22)). Detailed findings from this study have been previously reported in Camidge 2021 [15].

In the ALTA-1L trial, a one-sided cross-over from the control treatment to the experimental treatment was endorsed by the protocol. Patients assigned to receive crizotinib were permitted to crossover to the brigatinib arm after experiencing disease progression. Notably, 47% of participants who were randomized to crizotinib (65 out of 138) crossed over to brigatinib. The lack of a significant survival advantage for brigatinib, despite promising PFS results, may be attributed to treatment crossover, which may have reduced the statistical power of the usual ITT analysis [12, 13] and underestimated the benefits of brigatinib. Two sensitivity analyses that adjusted for confounding from crossover [15] suggested that brigatinib may have improved OS compared to crizotinib if crossover had not been allowed. Results from such analyses reported an HR of 0.50 (0.28–0.87) by the Inverse Probability of Censoring Weights (IPCW) method and 0.54 (0.31–0.92) by a Marginal Structural Model (MSM) method.

#### Choosing a causal estimand

Our re-analysis estimates the causal effect of brigatinib versus crizotinib on OS in advanced NSCLC patients, had control group patients not switched treatment and all trial participants remained under follow-up. Specifically, in our analyses we apply the IPCW approach [9, 12, 16, 17] to reproduce the ALTA-1L OS results and explore the sensitivity of this approach to different model specifications. We also apply Robins’ parametric g-formula [11, 18, 19] as a sensitivity analysis.

Our paper is structured as follows. In the Methods section, we motivate the need for the use of IPCW and the parametric g-formula to address treatment switching in the ALTA-1L trial using a Directed Acyclic Graph (DAG). We then describe the methods employed and their assumptions. In the Results section, we present and compare our findings across different methods, supported by various sensitivity analyses. In the Discussion section, we evaluate and compare the different approaches employed in our analysis, the plausibility of assumptions and the trade-offs addressing their interpretation in the context of the ALTA-1L trial. We also highlight the limitations inherent in our analysis, acknowledging the challenges and potential sources of bias associated with the methods utilized. We then conclude our paper by offering a road map for the use of IPCW and g-formula in similar research settings.

## Methods

### Data structure and notation

The ALTA-1L trial data consists of regular measurements of the following treatment, covariates, and outcome. The Treatment variable (*A*) in Time Interval (*k*), denoted as $A_k$, is an indicator of receiving brigatinib (versus crizotinib). Additional columns in the data set index a set of Time-Varying Covariates (TVCs) with values 0 or 1 indicating the less and more severe states respectively for Disease Progression (DP) and Intracranial Disease Progression (ICP). The categorized Eastern Cooperative Oncology Group (ECOG) score (ECOG) and continuous Target-Lesion Size (TLS) were also measured over time. Variables of interest changed at 30-day intervals reflecting the Follow-Up Visits (FUT). The Randomized Treatment Assignment (*Z*) and the following time-fixed baseline covariates are also available: Age (AGE), ECOG score (ECOG), Measurable Intracranial Central Nervous System (CNS) disease (ICS), Race (RACE), Sex (SEX), Smoking History (SM), Strata at randomization (baseline brain metastases and previous chemotherapy) (ST), Initial Diagnosis Stage (IDS), Lung Involvement at Study Entry (LI), and Prior Radiation Therapy (RT). We denote all TVCs as $L_{k}$, where $L_0$ includes baseline covariates. The outcome $Y_{k+1}$ is an indicator of death by the end of interval $k+1$. The variable $C_{k+1}$ is an indicator of censoring due to LTFU or Administrative Censoring (AC) by the end of the interval $k+1$. We use overbars to denote the history of the variable (i.e., $\bar{A}_{k} = (A_0, A_1, \dots , A_{k})$).

The data obtained from the ALTA-1L trial includes protected health information, and is therefore only accessible under controlled access procedures. To illustrate the data structure, we have constructed a synthetic version of individual-level data that reflects the relationships among a selected group of variables. The R code used to create the synthetic data set and implement the different analysis methods (IPCW and g-formula) is available in the following GitHub repository. A data dictionary can also be found in the Electronic Supplementary Material (ESM) 1.

### Treatment strategies and causal estimands

For the ITT analysis, we define the two treatment strategies as follows:

- “Assign to crizotinib” (${Z} ={0}, \bar{C} = \bar{0}$): This strategy assigns crizotinib at baseline and enforces no LTFU.
- “Assign to brigatinib” (${Z} ={1}, \bar{C} = \bar{0}$): This strategy assigns brigatinib at baseline and enforces no LTFU.

Our analyses focus on the following two hypothetical treatment strategies concerning drug intake:

- “Always treat with crizotinib” ($\bar{A} = \bar{0}, \bar{C} = \bar{0}$): This strategy administers crizotinib at each time point and enforces no LTFU.
- “Always treat with brigatinib” ($\bar{A} = \bar{1}, \bar{C} = \bar{0}$): This strategy administers brigatinib at each time point and enforces no LTFU.

For the purpose of this paper, we refer to the analyses under the latter treatment strategies as Per-Protocol (PP) analyses.

For the PP analyses, we aim to estimate the causal effect of “always treat with brigatinib” versus “always treat with crizotinib” on OS in advanced NSCLC patients, had control group patients not switched treatment and all trial participants remained under follow-up. To express our estimand, we utilize superscripts to represent counterfactual variables in our study. Specifically, $Y_{k+1}^{\bar{a},\bar{c} = \bar{0}}$ signifies the outcome for an individual at time $k+1$ if they had followed treatment strategy $\bar{a}$ assigned to them at baseline and censoring was eliminated. The counterfactual risk $Pr\left[ Y_{k+1}^{\bar{a},\bar{c}= \bar{0}} = 1\right]$ denotes the risk (cumulative incidence) at time $k+1$ under a joint intervention where treatment is set to $\bar{a}$ and censoring is eliminated. Our estimands include the counterfactual risks at each month of follow-up and relative risk at month 48 under the two intervention strategies.

### Drawing the directed acyclic graph (DAG)

**Fig. 1:**
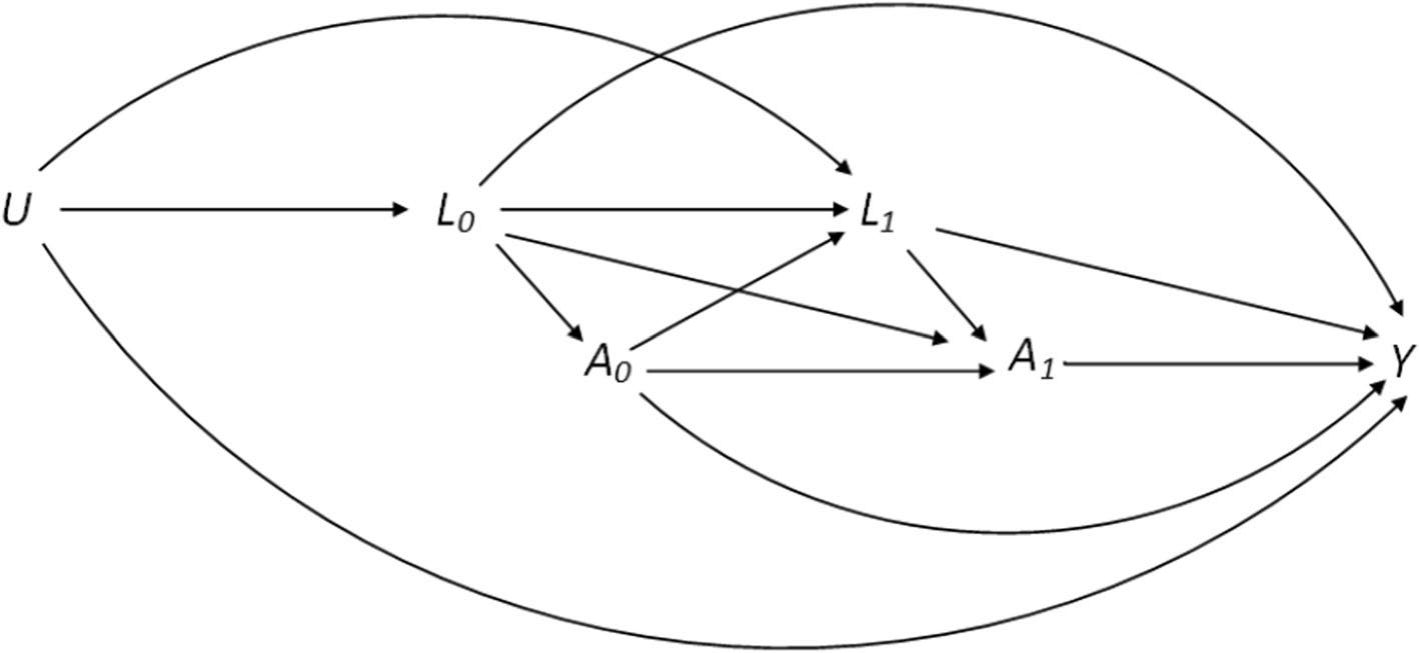
Graphical representations of our research focus and the bias of naive methods for time-varying confounding in the presence of treatment-confounder feedback: This Directed acyclic graph (DAG) represents the post-progression period for patients in the control arm. Following disease progression, the decision as to whether or not a patient in the control group continues to take its treatment ($A_{0}$) or ($A_{1}$) depends on predictive factors measured at or before the time of disease progression ($L_{0}$). Again, we assume that treatment intake ($A_{0}$) affects outcome (*Y*) and influences a set of TVCs at a later time-point ($L_{1}$). These covariates are also assumed to impact the clinician’s decision as to whether or not a patient should subsequently change treatments ($A_{1}$) introducing time-varying confounding and treatment-confounder feedback through $L_{1}$

Prior to discussing our applied methods, we visually depict the clinical setting of the ALTA-1L trial and motivate the use of g-methods in Fig. 1 using a DAG. For simplicity, we restrict our DAG to the post-progression period for patients in the crizotinib arm and consider two follow-up time points. Arrows between variables were placed based on domain knowledge and expert opinions as documented in Camidge et al [15]. Our DAG serves as a structural tool for better visualisation of the relationships between the following variables involved in the causal relationship of interest: baseline and TVCs ($L_{k}$), treatment intake ($A_{k}$) and outcome (*Y*). Additionally, we include in our DAG a vector of unmeasured covariates (*U*), which influences both the outcome and treatment. However, we rely on the strong and untestable assumption, that the effects of all unmeasured confounders (*U*) are adequately captured through the measured covariates ($L_{k}$). We deem this assumption reasonable for the ALTA-1L trial, given the availability of rich data on measured covariates at progression and throughout follow-up. Failure to account for $L_{1}$ in the analysis results in $L_{1}$ confounding the association between $A_{1}$ and *Y*. Adjustment methods that condition on $L_{1}$ may introduce two biases as follows: (i) Over-adjustment bias where the causal path $A_{0} \rightarrow L_{1} \rightarrow Y$ becomes blocked and (ii) Collider bias where the blocked path $A_{0} \rightarrow L_{1} \leftarrow \text {U} \rightarrow Y$ by the collider $L_{1}$ is opened. While the former bias removes part of the effect of the previous intake of treatment ($A_{0}$) on the outcome (*Y*) that is mediated by covariates at the following visit ($L_{1}$), the latter opens an initially blocked path and introduces an association between $A_{0}$ and *Y*. To address this dilemma, we employ g-methods that are capable of handling time-varying confounding affected by past exposure to estimate the treatment effect in the absence of LTFU and treatment switching.

### Survival analysis from discrete-time data

Recall that our data is structured in discrete-time format, where we define a time-varying indicator for death by the end of month *k*, $Y_{k}$. The discretization facilitates the modelling of complex relationships between time-varying variables and survival, while, for small intervals, the discrete-time format and corresponding analyses provide a close approximation to the event-process in continuous time. Here, we briefly review some foundations in survival analyses from discrete-time data and refer readers to Chapter 1.2 in the book *Modeling Discrete Time-to-Event Data* by Tutz and Schmid [20], and Chapter 17 of the *What If* book by Hernán [21] for further details.

Our analyses focus on estimating survival curves from discrete-time data. The discrete-time hazard at month k is defined as $Pr[Y_k = 1 \mid Y_{k-1} = 0]$. The survival probability at the end of month k, $Pr[Y_k = 0]$, can be expressed in terms of the discrete-time hazards by:

$$\begin{aligned} Pr[Y_k = 0] = \prod _{m=1}^{k} Pr[Y_m = 0 | Y_{m-1} = 0] \end{aligned}$$

One can then estimate the discrete-time hazard at month *k* by dividing the number of deaths during month *k* by the number at individuals alive at the end of month $k-1$. Plugging in this estimator of the discrete-time hazard results in the Kaplan-Meier estimator of the survival curve. Alternatively, one can use a logistic regression model to estimate the discrete-time hazard (e.g., including polynomial terms for time *k*). Many of the estimators we consider in this work involve estimating $Pr[Y_k = 1 \mid X = x]$ for a set of covariates X, and we consider logistic regression models to estimate discrete-time hazards $Pr[Y_k = 1 \mid Y_{k-1} = 0, X=x]$.

Apart from survival probabilities, we also consider the so-called hazard ratio (HR), discrete hazard ratio (dHR) and cumulative hazard ratio (cHR), which are discussed in detail in ESM 2.

### Methods used to estimate the causal estimands

In Table 1 we present a high-level summary of the different approaches we employ in our analyses. With the exception of the parametric g-formula approach, we applied the following methods to estimate the different causal estimands: (i) Non-parametric Kaplan Meier (KM) estimator to estimate counterfactual risks, Cumulative Hazard Ratios (cHRs) and Risk Ratios (RRs) (ii) Pooled logistic regression model to estimate counterfactual risks, Discrete Hazard Ratios (dHRs), cHRs and RRs and (iii) CoxPH regression model to estimate HRs. For pooled logistic regression analysis we used the whole dataset and included time (numbered from 0 to 48 and occurred every one month) as a covariate to specify a functional form for the baseline hazard. To allow time to be included in a more flexible manner, we have included time as a quadratic polynomial. Reported CIs across the different approaches were calculated using a non-parametric bootstrap procedure based on 1000 samples.

**Table 1:** Summary of methodology

Method		Summary Description	Assumptions
**Intention to treat**		Utilizes data from all randomized patients to estimate the causal effect of treatment assignment on the outcome.	No unmeasured confounding for treatment assignment and censoring, positivity, consistency, no model misspecification, and no measurement error
**Per protocol**	Excluding switchers	Patients who were initially randomized to the control arm but later received the experimental treatment were entirely excluded from the analysis.	No unmeasured confounding for treatment intake, non-informative censoring due to LTFU/AC, positivity, consistency, correct model specification, and no measurement error
	Censoring at switching	Patients who were randomized to the control arm but later received the experimental treatment were censored at the time of switching.	
	IPCW	Every time a control patient crosses over to the experimental treatment or is LTFU, this patient is censored at that time and the remaining similar control patients (in terms of specified baseline and TVCs) count for more patients to represent that patient. The remaining control patients are up-weighted to efficiently construct a pseudo-population that has the same definite characteristics as the original population but did not cross over or LTFU	No unmeasured confounding for treatment intake and censoring, positivity, consistency, correct model specification, and no measurement error
	Parametric g-formula	The parametric g-formula estimates the counterfactual risk of the treatment strategy at each time point by performing Monte Carlo simulation based on the fitted models of treatment, outcome and confounders	

*Abbreviations:* LTFU Lost to follow-up, *AC* Administratively censored, *TVC* Time-varying covariate, *IPCW *Inverse probability of censoring weights

The cumulative incidence at each visit is calculated as 1 minus the estimated survival function under that treatment strategy.

### Intention to treat analysis

Recall that conventional ITT analyses under the treatment strategy estimate the causal effect of treatment assignment on the outcome. In this type of analysis, censoring is employed for patients who are lost to follow-up.

In our first set of approaches, we assume non-informative censoring. Specifically, we did not adjust for any confounders. We also included an approach that accounted for strata at randomization, as well as adjusting for baseline confounders [22] where we considered the same set of baseline covariates previously described in Data structure and notation section. We also estimated marginal causal estimands (cHR and RR) under the treatment strategy standardized over baseline covariates after estimating the baseline hazard using a pooled logistic regression [23]. This was done by creating two new datasets each including a copy of every person at baseline and forcing everyone to be assigned to crizotinib in one data set and to brigatinib in the other.

To relax the assumption of non-informative censoring, we apply the IPCW approach to adjust for LTFU/AC. Since our IPCW approach in the PP analyses includes weighting to adjust for LTFU/AC, we refer readers to Per protocol analysis section for additional details on our implementation of this approach. In Tables S1 and S2 in ESM 3 we present details about the different model specifications for LTFU/AC.

### Per protocol analysis

#### Naive adjustment methods: Excluding or censoring patients at switching

In the existing literature [1, 6, 24–26], a range of approaches has been described to address situations where participants deviate from their assigned treatments. Approaches, commonly referred to as naive methods [27], typically involve minor modifications to standard survival techniques. In this section, we will concentrate on two methods: excluding patients who switch treatments and censoring patients at the time of switching.

Here we performed two distinct analyses where patients initially randomized to the control arm but later received the experimental treatment were either (i) entirely excluded from the analysis or (ii) their data censored at the time of switching. In these analyses, we assume that patients remain comparable irrespective of switching or LTFU. Although this assumption is straightforward, recall that it does not align with our assumptions depicted in Fig. 1 due to the presence of treatment-confounder feedback. We conduct such analyses (with and without adjustment for baseline covariates) to illustrate the inherent risk of selection bias and confounding. For the baseline adjusted analyses, we considered the same set of covariates previously described in Data structure and notation section.

#### Inverse probability of censoring weights

IPCW [12] can properly adjust for confounding and selection bias in the presence of treatment-confounder feedback [16]. Under the five different assumptions of no unmeasured confounding, positivity, consistency, correct model specification, and no measurement error [16], IPCW reconstructs a “representative sample” of the original cohort, often referred to as a “pseudo-population”, wherein censoring events become independent of any measured covariates [16]. Referring to our DAG in Fig. 1 and to account for treatment switching in the ALTA-1L, this means removing all arrows from $L_{k}$ to $A_{k}$. This is achieved by deferentially assigning to each individual at each time interval a weight that is inversely proportional to the individual’s probability of remaining unswitched up to a given time point. This probability is unknown and needs to be estimated from the data depending on some of the participants’ baseline and TVCs.

We describe the process of IPCW that accounts for treatment switching in the ALTA-1L trial as follows: Every time a control patient crosses over to the brigatinib treatment following disease progression, this patient is censored at that time and the remaining similar control patients (in terms of specified baseline and TVCs) count for more patients to represent that patient. Technically, the remaining control patients are up-weighted to efficiently construct a pseudo-population that has the same definite characteristics as the original population but did not cross over [28]. Adequate variability in the cross-over pattern should exist where everyone could be possibly exposed within levels of the time-varying and baseline covariates, otherwise, the weights cannot be defined [28]. For example, IPCW would not have been applicable in our analysis if all participants who developed disease progression crossed over to brigatinib.

Similarly, when accounting for LTFU/AC, the process is repeated. Each time a patient is lost to follow-up/administratively censored, that patient is censored, and the remaining patients who exhibit similar characteristics are up-weighted to construct a pseudo-population that mirrors the definitive characteristics of the original population but has remained under follow-up[28].

In our analysis, we focus on a joint intervention where both censoring events (switching and LTFU) are eliminated. To account for these two distinct events, we construct two separate weights. The resulting final pseudo-population is a result of the product of these two weights. In such a pseudo population, we then regress the outcome on the exposure using a standard regression model that does not include the measured confounders as covariates.

##### Weighting model

Following Robins and Finkelstein’s work [12], we estimated the weights for treatment switching and LTFU/AC separately in each treatment arm. Starting with switching weights, we leveraged our deterministic understanding of the known relationships between the randomized group, disease progression and switching status over time. Specifically, for all patients in the brigatinib arm, as well as patients who did not progress or before having progression in the crizotinib arm, a weight of 1 for switching was assigned to them at all time points. To estimate the probability of switching in the crizotinib arm after progression and derive the corresponding weights, we employed two distinct weighting models. The first pooled regression model for the denominator of the weights estimates the probability of switching at each time point, accounting for baseline and time-varying confounders. The second pooled logistic regression model estimates the probability of switching based on time alone (linear or splines) which is used for the numerator to stabilize the weights.

We performed eight analyses to adjust for treatment switching. Below, we outline the eight specifications for calculating the weights in the crizotinib arm. The weighting model for the denominator was fitted to the data after disease progression. We only considered variables in the weighting model that were potential confounders based on domain knowledge and expert opinions as documented in Camidge et al. 2021 [15]. Due to the limited number of patients in the trial, we performed variable selection amongst these potential confounders using criteria based on Akaike information criteria, a *p*-value of 0.20 or lower, or an HR or Odds Ratio (OR) less than 0.75 or greater than 1.33 for death or switching. This selection process was only used to define specifications 1 and 2, which we detail next. The remaining specifications aim to either relax the linearity assumption or explore the influence of potential residual confounding within categories of the confounders. In describing the specifications, we adopt the notation that numerical variables are labeled as *variablename*$^n$, where *n* indicates the type of transformation (1: linear term, 2: quadratic term, *S*: spline transformation), and categorical variables are labelled as *variablename*$_n$, where *n* represents the number of categories (see Table S3 in ESM 4 for a summary).

- **Specification 1**: The weighting model for the denominator included variables that are prognostic of survival (full model) and incorporated the following variables: Linear terms for Follow-Up Time (FUT) and Target Lesion Size (TLS), linear and quadratic terms for Time-to-Disease Progression (TDP, TDP^2^) and a step function with 3 categories for time-varying ECOG (ECOG_3_). Baseline variables included a linear term for Age (AGE) and a step function with 2 categories for Measurable Intracranial CNS Disease Status (ICS_2_) and Smoking History (SM_2_), as well as a step function with 4 categories for Initial Diagnosis Stage (IDS_4_), Lung Involvement at Study Entry (LI_4_) and Strata at Randomization (ST_4_). In Table S4 in ESM 3 we provide the results from a multivariate pooled logistic regression analysis - full model - for the probability of switching conditional on baseline and TVCs in the control arm using post-progression data only. To estimate the numerator of the weights, we employed another pooled logistic model, for the probability of switching based on a linear term for time.
- **Specification 2**: The weighting model for the denominator included variables that jointly predict switching and survival (restricted model). These variables were selected from the list of covariates included in specification 1. The model included the following TVCs: FUT, TLS, TDP, and TDP^2^. Baseline variables included AGE, IDS_4_, and ST_4_. In Table S5 in ESM 3 we provide the results from a multivariate pooled logistic regression analysis - restricted model - for the probability of switching conditional on baseline and TVCs in the control arm using post-progression data only. To estimate the numerator of the weights, we employed another pooled logistic model, for the probability of switching based on a linear term for time.
- **Specification 3**: Similar to specification 1, this specification included the same variables but with 5 knots splines for time (FUT^S^), time to disease progression (TDP^S^), target lesion size (TLS^S^) and baseline age (AGE^S^). This approach helps mitigate potential bias stemming from strong linearity assumptions in the model.
- **Specification 4**: Similar to specification 1, but replaces ECOG_3_ with 2 categories ECOG_2_. This allows us to explore the influence of potential residual confounding within categories of the confounders.
- **Specification 5**: Similar to specification 2, but replaces the linear and quadratic terms with FUT^S^, TDP^S^, TLS^S^ and AGE^S^.
- **Specification 6**: Similar to specification 2, but excluding IDS_4_ as it weakly correlates with switching.
- **Specification 7**: Similar to specification 1, but excluding TDP and TDP^2^.
- **Specification 8**: Similar to specification 2, but excluding TDP and TDP^2^.

By employing these models, we generated predicted probabilities for each individual at each time point, which were subsequently used to calculate the inverse probability of switching weights. Unstabilized weights were constructed as the inverse of the denominator, while stabilized weights were calculated as the ratio of the numerator over the denominator.

The same process was repeated to adjust for LTFU/AC by fitting pooled logistic regression models separately for each treatment arm to estimate the probability of LTFU/AC and derive the corresponding weights. For details about each specification, we present in Tables S1 and S2 in ESM 3 the results from a multivariate pooled logistic regression analysis - full and restricted model respectively - for the probability of LTFU/AC conditional on baseline and TVCs by randomized arm. LTFU/AC probabilities derived from this analysis are used to calculate the denominator of LTFU/AC weights.

##### Outcome model

To estimate the causal effect of “always treat with brigatinib” versus “always treat with crizotinib” on OS, we employed a weighted pooled logistic regression –with randomized treatment arm and linear and quadratic terms for time as predictors– to estimate dHRs, RRs and cHRs and a time-dependent CoxPH outcome model to estimate HRs. The dataset provided included all observations up to the minimum time of switching and/or LTFU/AC weighted by the product of the inverse probability of switching weights and LTFU/AC weights. The respective 95% CIs were estimated using bootstrapping based on 1000 samples to account for correlated data and the weight estimation process. cHRs and RRs from a KM estimator were also estimated. We also created weighted KM survival curves using the “survfit” function from the survival package in R and using the “weights” argument to apply the weights. The R code for implementing the IPCW analysis on the synthetic data is provided in the following GitHub repository.

#### Parametric g-formula

The (noniterative conditional expectation) parametric g-formula is another approach to estimate the effect of sustained treatment strategies from observational data [11, 29, 30]. Similar to IPCW, the parametric g-formula relies on the assumptions of no unmeasured confounding, positivity, consistency, correct model specification, and no measurement error. Initially introduced by Robins in 1986 [11], the parametric g-formula extends the concept of standardization to account for time-varying treatments. This approach involves fitting parametric models for the conditional discrete-time hazards of the outcome and joint conditional density of the confounders at each time point given prior treatment and covariates history. The counterfactual risk of the treatment strategy at each time point is estimated by performing Monte Carlo simulation based on the fitted models, which can be thought of as simulating the distribution of covariates, treatment, and outcome in a population of patients undergoing the given treatment strategy.

Here, we apply the parametric g-formula to the 2-arm ALTA-1L RCT to estimate the counterfactual mortality risk at each follow-up time point under the “always treat with brigatinib” strategy and “always treat with crizotinib” strategy. Each arm of the RCT was analysed and interpreted as a separate observational study, as trial participants who do not comply with their assigned treatment and are lost to follow-up may differ systematically from those who comply and remain under follow-up [31].

We considered the same baseline and post-randomization determinants of switching and LTFU as previously described. The baseline covariates include: age, ECOG score, measurable intracranial CNS disease, race, sex, smoking history, strata at randomization and prior radiation therapy. The two categorical baseline variables, initial diagnosis stage and lung involvement at study entry, were excluded from this analysis. These variables caused some of the bootstrap replicates to fail when constructing the CIs due to the limited number of observations within certain categories of these variables. However, the exclusion of these variables did not materially change the effect estimates (cHR and RR). We also considered the following TVCs: disease progression (binary), intracranial disease progression (binary), ECOG (binary and categorical), and target-lesion size (continuous). We used pooled over time logistic regression to model the binary and categorical TVCs and used pooled over time linear regression to model the continuous TVCs. We also used a pooled over time logistic regression model for the discrete hazard of death.

We incorporated deterministic knowledge of some of the variables in the parametric g-formula algorithm to help mitigate bias from model misspecification. Specifically, we incorporated the knowledge that the disease progression and intracranial disease progression variables will take on a value of 1 at all subsequent time points after initially taking on a value of 1. The fitted models are therefore limited to observations where the value of the covariate at the previous time point is 0. We also incorporated the restriction that the probability of death is 0 for individuals who have not yet progressed.

We performed five different analyses with different model specifications for the TVCs and outcome. These five analyses broadly explore how more richly parameterizing the models can affect the causal effect estimates. In specification 1, for each fitted covariate and outcome model, we included all predictors with a *P*-value <0.2. Further refinements were applied in specifications 2 and 3, narrowing the selection to covariates with *P*-values <0.1 and 0.05, respectively. Specification 4 mirrored 1 but introduced three levels for the ECOG variable as opposed to the two levels. In specification 5, we removed time to progression from the outcome model. Specifications 4 and 5 were included to enable meaningful comparisons with the IPCW approach. A detailed description of the covariates included in each analysis is available in Tables S6 to S8 in ESM 5.

In each of our analyses, we estimated the counterfactual risk of each treatment strategy at each time point. We also estimated the RR and cHRs comparing the two treatment strategies. We constructed 95% CIs around the counterfactual risks, RRs, and cHRs using a non-parametric bootstrap procedure based on 500 samples.

We used the publicly available software in R (gfoRmula) [19] to perform the parametric g-formula analyses. The R code for performing the g-formula analysis on the synthetic data is provided in the following GitHub repository.

## Results

### Study population

The baseline characteristics of the intention-to-treat study population, comparing brigatinib to the crizotinib arm, were previously documented in Camidge 2018 [14]. Table 2 compares the baseline characteristics between switchers and non-switchers in the control group. Switchers seem younger and have higher target lesion sizes than non-switchers, but the remaining baseline variables seem very similar across the two groups.

**Table 2:** Distribution of baseline patient characteristics among switchers and non-switchers in the control group, ALTA-1L trial

Characteristics		Control, switchers	Control, non-switchers	*P*-value^a^
No. (% of patients in the control arm)		65 (47.1)	73 (52.9)	
Age in yr - Median (range)		56 (30.0,79.0)	62 (29.0,89.0 )	0.028
Female sex - No. (%)		34 (52.3)	47 (64.4)	0.206
Race - No. (%)	Non-Asian	38 (58.5)	51 (69.9)	0.223
	Asian	27 (41.5)	22 (30.1)	
ECOG performance-status score - No. (%)	0	25 (38.5)	28 (38.4)	0.593
	1	38 (58.5)	40 (54.8)	
	2	2 (3.1)	5 (6.8)	
History of tobacco use - No. (%)	Never smoked	36 (55.4)	39 (53.4)	0.601
	Former Smoker	27 (41.5)	29 (39.7)	
	Current Smoker	2 (3.1)	5 (6.8)	
Initial cancer diagnosis stage - No. (%)	IA	1 (1.5)	2 (2.7)	0.533
	IB	0 (0)	2 (2.7)	
	IIA	2 (3.1)	4 (5.5)	
	IIIA	5 (7.7)	9 (12.3)	
	IIIB	5 (7.7)	7 (9.6)	
	IV	52 (80.0)	49 (67.1)	
Histologic type - No. (%)	Adenocarcinoma	64 (98.5)	73 (100)	0.954
	Adenosquamous carcinoma	1 (1.5)	0	
	Squamous-cell carcinoma	0	0	
	Large-cell carcinoma	0	0	
Measurable intracranial CNS disease - No. (%)		10 (15.4)	13 (17.8)	0.879
Previous intake of anti-cancer therapy - No. (%)		20 (30.8)	23 (31.5)	1
Previous radiation therapy - No. (%)		18 (27.7)	22 (30.1)	0.898
Lung involvement at study entry - No. (%)		13 (20)	22 (30.1)	0.574
		22 (33.8)	21 (28.8)	
		25 (38.5)	24 (32.9)	
		5 (7.7)	6 (8.2)	
Target lesion size - Median (range)		51 (9.0,170.0)	38 (7.0,214.0 )	0.029
Brain metastasis - No.(%)		18 (27.7)	22 (30.1)	0.898
Previous chemotherapy - No.(%)		20 (30.8)	17 (23.3)	0.425

*Abbreviations:* *ECOG* Eastern cooperative oncology group, *CNS *Central nervous system

^a^Differences in baseline characteristics between switchers and non-switchers were evaluated using a two-sample t-test for continuous data and a chi-square test for categorical data

### Outcomes

Previous reports [14, 15] have already provided detailed results for the primary endpoint (PFS) from the ALTA-1L trial and other secondary endpoints. Our analysis focuses on OS as the endpoint of interest. The resulting data set includes 8624 person-months (4345 in the crizotinib arm and 4279 in the brigatinib arm). The total number of individuals in the ALTA-1L is 275 with 138 randomized to crizotinib and 137 randomized to brigatinib. For our switching adjusted analysis, we explored five different approaches: ITT, “naive methods” (excluding switchers and censoring at switching) and “g-methods” (IPCW and parametric g-formula).

**Fig. 2:**
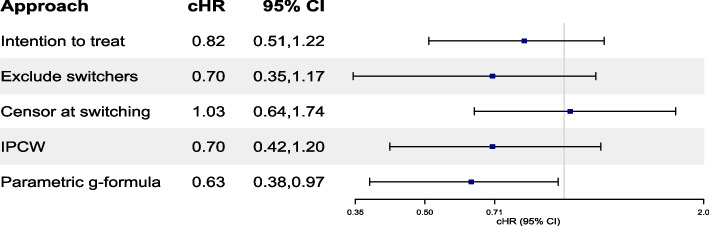
Forest plot of cHR and their 95% CI for the effect of brigatinib versus crizotinib on OS investigated through various modelling approaches. cHRs for the ITT, exclude switchers and censor at switching analyses were estimated from a pooled logistic regression model. cHR for the IPCW approach was estimated using a weighted pooled logistic regression model using the product of the two weights for LTFU/AC (specification 4 in Table 4) and switching (specification 4 in Table 5). cHR from the parametric g-formula were estimated from specification 1 in Table 7. 95% CIs were estimated using a non-parametric bootstrap procedure based on 1000 samples for all approaches and 500 samples for the parametric g-formula. cHR, cumulative hazard ratio; CI, confidence interval; OS, overall survival, IPCW, inverse probability of censoring weights; LTFU, loss to follow up; AC, administrative censoring

In Tables 3, 4, 5, 6, and 7, we present the results of all OS analyses. Table 3 offers a comprehensive summary across all approaches and reports HRs from a CoxPH, dHR, cHR and RR from a pooled logistic regression, and cHR and RR from a non-parametric KM estimator. Reported (95%CIs) were calculated using a non-parametric bootstrap procedure based on 500 or 1000 samples. In Table S9 in ESM 6, we outline the number of replicate failures observed in each analysis. Findings from Table 3 are also visualized through a forest plot as shown in Fig. 2 and Figure S1 in ESM 7.

**Table 3:** Summary of results: Effect of brigatinib versus crizotinib on Overall Survival (OS) investigated through various approaches; ALTA-1L trial

Approach		CoxPH Model	Pooled Logistic Regression Model			Kaplan-Meier Estimator	
		HR^a^ (95% CI)^e^	dHR^b^ (95% CI)^e^	cHR^c^ (95% CI)^e^	RR^d^ (95% CI)^e^	cHR^c^ (95% CI)^e^	RR^d^ (95% CI)^e^
**Intention to Treat**	Unadjusted for baseline covariates	0.82 (0.52,1.22)	0.82 (0.51,1.22)	0.82 (0.51,1.22)	0.86 (0.59,1.17)	0.83(0.51,1.28)	0.86 (0.59,1.21)
	Adjusted for strata at randomization^g^	0.80 (0.50,1.21)	0.79 (0.49,1.21)	0.89 (0.56,1.37)	0.91 (0.63,1.29)	NA	NA
	Adjusted for baseline covariates^h^	0.79 (0.44,1.32)	0.79 (0.44,1.33)	0.72 (0.30,1.76)	0.76 (0.35,1.65)	NA	NA
	Marginal effect adjusted for baseline covariates	NE	NE	0.84 (0.54,1.25)	0.86 (0.59,1.22)	NA	NA
**Per Protocol**	Excluding switchers	0.70 (0.36,1.17)	0.70 (0.35,1.17)	0.70 (0.35,1.17)	0.76 (0.43,1.13)	0.77 (0.37,1.27)	0.82 (0.46,1.21)
	Censoring at switching	1.03 (0.64,1.73)	1.03 (0.64,1.74)	1.03 (0.64,1.74)	1.02 (0.69,1.58)	1.07 (0.64,1.96)	1.06 (0.70,1.73)
	Inverse probability of censoring weights^i^	0.71 (0.42,1.21)	0.70 (0.41,1.20)	0.70 (0.42,1.20)	0.77 (0.52,1.16)	0.70 (0.39,1.31)	0.76 (0.50,1.23)
	Parametric g-formula	NE	NE	0.63 (0.38,0.97)^f^	0.72 (0.50,0.98)^f^	NE	NE

*Abbreviations: CoxPH *Cox proportional hazard, *CI *Confidence interval, *NE *Not estimated, *NA *Not applicable

^a^ HR: Hazard ratio estimated based on a Cox proportional hazard model

^b^ dHR: Discrete hazard ratio estimated based on a pooled logistic regression model (refer to Electronic Supplementary Material (ESM) 2 for more details)

^c^ cHR: Cumulative hazard ratio by month 48 (equation (5) in ESM 2)

^d^ RR: Risk ratio formulated as the ratio of the cumulative risks by month 48

^e^ 95% CI calculated using a non-parametric bootstrap procedure based on 1000 samples

^f^ Reported CI calculated using a non-parametric bootstrap procedure based on 500 samples

^g^Strata at randomization: presence or absence of baseline brain metastases and completion of at least one full cycle of chemotherapy for locally advanced or metastatic disease (yes or no)

^h^Baseline covariates: age, ECOG score, measurable intracranial CNS disease, race, sex, smoking history, strata at randomization, initial diagnosis stage, lung involvement at study entry and prior radiation therapy

^i^Inverse probability of censoring weight: Estimates for the IPCW approach were estimated using a weighted pooled logistic regression model using the product of the two weights for LTFU/AC (specification 4 in Table 4) and switching (specification 4 in Table 6)

**Table 4:** Causal effect of “assign to brigatinib” versus “assign to crizotinib” on Overall survival (OS) investigated through various models (specifications 1 to 6) for the construction of inverse probability of censoring weights for Lost to follow-up (LTFU)/administrative censoring (AC); Death censored by LTFU/AC only; 92 deaths, ALTA-1L trial

Specification	Description	Estimated stabilized weights		Difference in OS	
		Mean (SD)	Min (Max)	cHR^b^ (95% CI)^d^	RR^c^ (95% CI)^d^
1	Full model ^a^	0.99 (0.15)	0.11 (6.45)	0.84 (0.53,1.26)	0.87 (0.61,1.20)
2	Restricted model ^a^	0.99 (0.15)	0.15 (6.66)	0.82 (0.52,1.23)	0.86 (0.60,1.17)
3	Same as specification 1, but replace the linear terms of time, target lesion size and age with 5 knots splines	0.99 (0.18)	0.09 (10.61)	0.83 (0.52,1.24)	0.87 (0.61,1.18)
4	Same as specification 1, but replace the step function (3 categories) for time-varying ECOG with 2 categories	0.99 (0.16)	0.11 (6.53)	0.83 (0.52,1.25)	0.87 (0.61,1.19)
5	Same as specification 2, but replace the linear terms of time, target lesion size and age with 5 knots splines	0.99 (0.18)	0.10 (10.30)	0.81 (0.50,1.21)	0.85 (0.59,1.15)
6	Same as specification 2, but remove baseline covariates: sex, baseline ECOG and initial diagnosis stage in calculating the denominator of the experimental arm	0.99 (0.17)	0.16 (10.27)	0.84 (0.53,1.27)	0.88 (0.62,1.20)

^a^Details about the full and restricted model are available in Tables S1 and S2 in the Electronic Supplementary Material (ESM) 3). Refer to Table S3 in ESM 4 for a full list of variable abbreviations used in this figure. $\text {Spec1}_{control}$: $\text {AGE}, \text {AGE}^2, \text {IDS}_{4}, \text {ICS}_{2}, \text {LI}_{4},\text {ST}_{4}, \text {SM}_{2}, \text {FUT}, \text {FUT}^2,\text {ECOG}_{3}, \text {TLS}, \text {DP}_{2}, \text {A}_{2}$. $\text {Spec1}_{exp}$: $\text {AGE}, \text {AGE}^2, \text {SEX}_{2}, \text {RACE}_{2}, \text {IDS}_{4}, \text {ICS}_{2}, \text {LI}_{4}, \text {ECOG}_{2}, \text {ST}_{4}, \text {SM}_{2}, \text {RT}_{2}, \text {FUT}, \text {FUT}^2, \text {ECOG}_{3}, \text {TLS},\text {IDP}_{2}, \text {DP}_{2}$. $\text {Spec2}_{control}$: $\text {AGE}, \text {AGE}^2, \text {IDS}_{4}, \text {ST}_{4}, \text {FUT}, \text {FUT}^2, \text {TLS}, \text {DP}_{2}$. $\text {Spec2}_{exp}$: $\text {AGE}^1, \text {AGE}^2, \text {SEX}_{2}, \text {IDS}_{4}, \text {ECOG}_{2}, \text {SM}_{2}, \text {FUT}, \text {FUT}^2, \text {ECOG}_{3}, \text {IDP}_{2}, \text {DP}_{2}$. Spec 3: Same as Specification 1, but with $\text {FUT}^S, \text {TLS}^S,\text {AGE}^S$ instead of linear terms. Spec 4: Same as Specification 1, but with $\text {ECOG}_{2}$ instead of $\text {ECOG}_{3}$. Spec 5: Same as Specification 2, but with $\text {FUT}^S,\text {TLS}^S,\text {AGE}^S$ instead of linear terms. Spec 6: Same as Specification 2, but without $\text {SEX}_{2}, \text {ECOG}_{2}, \text {IDS}_{4}$ in calculating the denominator of the experimental arm

^b^ cHR: Cumulative hazard ratio by month 48 (equation (5) in ESM 2)

^c^ RR: Risk ratio formulated as the ratio of the cumulative risks by month 48

^d^ Reported 95% Confidence Interval (CI) estimated using a non-parametric bootstrap procedure based on 1000 samples

**Table 5:** Causal effect of “always treat with brigatinib” versus “always treat with crizotinib” on Overall Survival (OS) investigated through various models (specifications 1 to 8) for the construction of the inverse probability of censoring weights for switching; Lost to follow-up (LTFU) assumed at random; Death censored by a minimum of treatment switching and LTFU/administrative censoring; 72 deaths, ALTA-1L trial

Specification	Description	Estimated stabilized weights		**Difference in OS**	
		Mean (SD)	Min (Max)	cHR^b^ (95% CI)^d^	RR^c^ (95% CI)^d^
1	Full Model ^a^	0.96 (0.25)	0.43 (5.15)	0.69 (0.39,1.21)	0.75 (0.49,1.16)
2	Restricted Model ^a^	0.97 (0.25)	0.43 (5.73)	0.69 (0.41,1.21)	0.75 (0.51,1.16)
3	Same as specification 1, but with 5 knots splines for time, time to disease progression, target lesion size and baseline age	0.95 (0.27)	0.44 (6.05)	0.67 (0.38,1.26)	0.74 (0.49,1.20)
4	Same as specification 1, but replace the step function (3 categories) for time-varying ECOG with 2 categories	0.96 (0.24)	0.43 (5.32)	0.69 (0.39,1.21)	0.75 (0.49,1.16)
5	Same as specification 2, but with 5 knots splines for time, time to disease progression, target lesion size and baseline age	0.96 (0.30)	0.44 (7.90)	0.65 (0.32,1.22)	0.72 (0.43,1.17)
6	Same as specification 2, but without step function (4 categories) for initial diagnosis stage	0.97 (0.24)	0.43 (3.39)	0.73 (0.45,1.22)	0.79 (0.54,1.17)
7	Same as specification 1, but without linear and quadratic terms for time to disease progression	1.09 (1.19)	0.43 (27.27)	0.40 (0.11,1.20)	0.53 (0.32,1.16)
8	Same as specification 2, but without linear and quadratic terms for time to disease progression	1.09 (1.06)	0.43 (25.86)	0.38 (0.12,0.98)	0.52 (0.32,0.98)

^a^Details about the full and restricted model are available in Tables S4 and S5 in the Electronic Supplementary Material (ESM) 3. Refer to Table S3 in ESM 4 for a full list of variable abbreviations used in this figure. Spec 1: $\text {AGE}, \text {IDS}_{4}, \text {ICS}_{2}, \text {LI}_{4}, \text {ST}_{4}, \text {SM}_{2}, \text {FUT}, \text {ECOG}_{3}, \text {TLS}, \text {TDP}, \text {TDP}^2$. Spec 2: $\text {AGE}, \text {IDS}_{4}, \text {ST}_{4}, \text {FUT}, \text {TLS}, \text {TDP}, \text {TDP}^2$. Spec 3: Same as Spec 1 but with $\text {FUT}^S, \text {TLS}^S,\text {Age}^S,\text {TDP}^S$ instead of their linear terms. Spec 4: Same as Spec 1 but with $\text {ECOG}_{2}$ instead of $\text {ECOG}_{3}$. Spec 5: Same as Spec 2 but with $\text {FUT}^S$, $\text {TLS}^S$, $\text {Age}^S$, and $\text {TDP}^S$ instead of their linear terms. Spec 6: Same as Spec 2 but without $\text {IDS}_{4}$. Spec 7: Same as Spec 1 but without $\text {TDP}$ and $\text {TDP}^2$. Spec 8: Same as Spec 2 but without $\text {TDP}$ and $\text {TDP}^2$

^b^ cHR: Cumulative hazard ratio by month 48 (equation (5) in ESM 2)

^c^ RR: Risk ratio formulated as the ratio of the cumulative risks by month 48

^d^ Reported 95% Confidence Interval (CI) estimated using a non-parametric bootstrap procedure based on 1000 samples

**Table 6:** Causal effect of “always treat with brigatinib” versus “always treat with crizotinib” on Overall Survival (OS) investigated through specification 7 from Table 5 for the construction of the inverse probability of censoring weights for switching; Weights under progressive truncation; Lost to follow-up (LTFU) assumed at random; Death censored by a minimum of treatment switching and LTFU/administrative censoring; 72 deaths, ALTA-1L trial

Truncation Percentile	Estimated stabilized weights		Difference in OS	
Truncation percentiles	Mean (SD)	Min (Max)	cHR^a^ (95% CI)^c^	RR^b^ (95% CI)^c^
0,100	1.09 (1.19)	0.43 (27.27)	0.40 (0.11,1.20)	0.53 (0.32,1.16)
1,99	1.05 (0.72)	0.43 (6.71)	0.57 (0.39,1.16)	0.66 (0.49,1.13)
5,95	0.94 (0.20)	0.43 (1.43)	0.77 (0.52,1.35)	0.81 (0.59,1.28)
10,90	0.92 (0.15)	0.43 (1)	0.84 (0.53,1.40)	0.87 (0.61,1.32)
25,75	0.92 (0.15)	0.43 (1)	0.84 (0.53,1.40)	0.87 (0.61,1.32)
50,50^d^	0.92 (0.15)	0.43 (1)	0.84 (0.53,1.40)	0.87 (0.61,1.32)

^a^ cHR: Cumulative hazard ratio by month 48 (equation (5) in ESM 2)

^b^ RR: Risk ratio formulated as the ratio of the cumulative risks by month 48

^c^ 95% Confidence Intervals (CIs) estimated using a non-parametric bootstrap procedure based on 1000 samples

^d^ Truncating weights to the $50^\text {th}$ percentile corresponds to an unadjusted model

**Table 7:** Causal effect of “always treat with brigatinib” versus “always treat with crizotinib” on Overall Survival (OS) investigated through specifications 1 to 5 using the parametric g-formula, ALTA-1L trial

Specification^a^	Risk (95% CI) in control	Risk (95% CI) in experimental	cHR^b^ (95% CI)^d^	textbfRR^c^ (95% CI)^d^
**1**	0.56 (0.41,0.67)	0.41 (0.29,0.49)	0.63 (0.38,0.97)	0.72 (0.50,0.98)
**2**	0.56 (0.41,0.68)	0.39 (0.29,0.49)	0.61 (0.38,0.91)	0.71 (0.49,0.93)
**3**	0.52 (0.40,0.63)	0.39 (0.27,0.48)	0.68 (0.40,0.97)	0.75 (0.50,0.98)
**4**	0.53 (0.39,0.65)	0.42 (0.30,0.50)	0.72 (0.43,1.07)^e^	0.79 (0.54,1.05)^e^
**5**	0.55 (0.40,0.68)	0.39 (0.27,0.48)	0.62 (0.36,0.92)	0.71 (0.48,0.94)

^a^Details about Specifications 1, 2 and 3 are available in Tables S6, S7 and S8 in the Electronic Supplementary Material (ESM) 5. Specifications 4 and 5 are the same as Specification 1 but replacing 2 categories of ECOG with 3 categories for Specification 4 and without progression time for Specification 5

^b^cHR: Cumulative hazard ratio by month 48 based on a pooled logistic regression model (equation (5) in the ESM 2)

^c^RR: Risk ratio formulated as the ratio of the counterfactual risks by month 48

^d^95% CIs estimated using a non-parametric bootstrap procedure based on 500 samples

^e^95% CIs estimated using a non-parametric bootstrap procedure based on 500 samples with 60 replicate failures

### Results from the ITT analysis

A total of 92 deaths occurred (41 of 137 patients [30%] in the brigatinib arm and 51 of 138 patients [37%] in the crizotinib group). Using a KM estimator, the cumulative survival probability at 48 months was 60% in the crizotinib arm and 65% in the brigatinib arm. Figure S2a in ESM 7 displays the KM curve. The KM estimator yielded a cHR of 0.83 (0.51,1.28) and RR of 0.86 (0.59,1.21). Using a pooled logistic regression model, the reported cHR was 0.82 (0.51,1.22) and RR was 0.86 (0.59,1.17). A CoxPH regression analysis, considering only the treatment assignment as a dichotomous covariate, yielded an HR of 0.82 (0.52, 1.22). HRs from the CoxPH model were similar to the reported dHR from a pooled logistic regression.

ITT adjusted analysis by strata at randomization yielded a cHR of 0.89 (0.56, 1.37) and an RR of 0.91 (0.63,1.29) from a pooled logistic regression. When adjusting for all baseline covariates, the cHR and RR from a pooled logistic regression were 0.72 (0.30,1.76) and 0.76 (0.35,1.65). HRs from a CoxPH regression analysis and dHR from a pooled logistic regression did not materially change across the different analyses.

A marginal effect analysis, based on a pooled logistic regression adjusted for all baseline covariates, resulted in a cHR of 0.84 (0.54, 1.25), and an RR of 0.86 (0.59, 1.22). We also provide the KM curve in Figure S2b in ESM 7 for the probability of OS over time standardized for baseline covariate distribution.

**Fig. 3:**
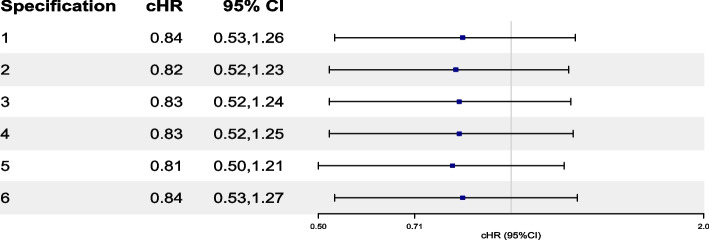
Forest plot of cHR and 95% CI for the causal effect of “assign to brigatinib” versus “assign to crizotinib” on OS investigated through specifications 1 to 6 using IPCW. Death is censored by LTFU/AC. Details about Specifications 1 (full model) and 2 (restricted model) are also available in Tables S1 and S2 in ESM 3. Refer to Table S3 in ESM 4 for a full list of variable abbreviations used in this figure. $\text {Spec1}_{control}$: $\text {AGE}, \text {AGE}^2, \text {IDS}_{4}, \text {ICS}_{2}, \text {LI}_{4},\text {ST}_{4}, \text {SM}_{2}, \text {FUT}, \text {FUT}^2,\text {ECOG}_{3}, \text {TLS}, \text {DP}_{2}, \text {A}_{2}$. $\text {Spec1}_{exp}$: $\text {AGE}, \text {AGE}^2, \text {SEX}_{2}, \text {RACE}_{2}, \text {IDS}_{4}, \text {ICS}_{2}, \text {LI}_{4}, \text {ECOG}_{2}, \text {ST}_{4}, \text {SM}_{2}, \text {RT}_{2}, \text {FUT}, \text {FUT}^2, \text {ECOG}_{3}, \text {TLS},\text {IDP}_{2}, \text {DP}_{2}$. $\text {Spec2}_{control}$: $\text {AGE}, \text {AGE}^2, \text {IDS}_{4}, \text {ST}_{4}, \text {FUT}, \text {FUT}^2, \text {TLS}, \text {DP}_{2}$. $\text {Spec2}_{exp}$: $\text {AGE}^1, \text {AGE}^2, \text {SEX}_{2}, \text {IDS}_{4}, \text {ECOG}_{2}, \text {SM}_{2}, \text {FUT}, \text {FUT}^2, \text {ECOG}_{3}, \text {IDP}_{2}, \text {DP}_{2}$. Spec 3: Same as Specification 1, but with $\text {FUT}^S, \text {TLS}^S,\text {AGE}^S$ instead of linear terms. Spec 4: Same as Specification 1, but with $\text {ECOG}_{2}$ instead of $\text {ECOG}_{3}$. Spec 5: Same as Specification 2, but with $\text {FUT}^S,\text {TLS}^S,\text {AGE}^S$ instead of linear terms. Spec 6: Same as Specification 2, but without $\text {SEX}_{2}, \text {ECOG}_{2}, \text {IDS}_{4}$ in calculating the denominator of the experimental arm. cHRs were estimated from a weighted pooled logistic regression using weights for LTFU/AC and formulated based on equation (5) provided in ESM 2. Reported CIs were estimated using a non-parametric bootstrap procedure based on 1000 samples. cHR, cumulative hazard ratio; CI, confidence interval; OS, overall survival, IPCW, inverse probability of censoring weights; LTFU, loss to follow up; AC, administrative censoring; ESM: electronic supplementary material

Results from our IPCW analyses that adjust for LTFU/AC (Table 4), resulted in cHRs that ranged between 0.81 (0.50,1.21) and 0.84 (0.53,1.27) using a weighted pooled logistic regression model. The estimated weights were relatively stable, with a mean of 0.99 and Standard Deviation (SD) between 0.15 and 0.18. Findings from Table 4 are also visualized through a forest plot in Fig. 3 and Figure S3 in ESM 7. In Table S10 in ESM 8 we report the summary statistics of unstabilized weights and corresponding effect estimates.

### Results from the PP analysis

#### Results from the naive adjusted analysis

In the approach where switchers were censored at the point of switching, a relatively high level of censoring was observed. Over 12, 24, 36, and 48 months, the number of individuals remaining in the control arm decreased from 137 to 84, 53, 42, and 2, respectively. In the remaining uncensored, a total of 70 deaths occurred (29 in the crizotinib group and 41 in the brigatinib group). Using a KM estimator, the cumulative survival probability at 48 months was 67% in the crizotinib arm and 65% in the brigatinib arm. The total follow-up time, while participants adhered to their assigned treatment in terms of switching, was 2865 person-months (66%) in the crizotinib arm and 4279 person-months (100%) in the brigatinib arm. Figure S4a in ESM 7 displays the KM curve of OS adjusted for treatment switching and Table 3 shows the reported KM results. The estimated cHR and RR for OS based on a pooled logistic regression comparing brigatinib to crizotinib when switchers were censored were 1.03 (0.64,1.74) and 1.02 (0.69,1.58) respectively with no adjustment for baseline covariates and 0.62 (0.20,2.67) and 0.67 (0.25,2.43) after adjusting for baseline covariates.

The estimated cHR and RR based on a pooled logistic regression model were 0.70 (0.35,1.17) and 0.76 (0.43,1.13) respectively when switchers were entirely excluded from the analysis. The estimated cHR and RR after adjusting for baseline covariates were 0.49 (0.11,2.17) and 0.55 (0.15,2.74) respectively. The cumulative survival probability at 48 months was 58% in the control arm and 65% in the treatment arm using KM estimator. Figure S4b in ESM 7 displays the KM curve of OS adjusted for treatment switching.

Additional results across different approaches and estimators are provided in Table 3.

#### Results from the IPCW analysis

**Fig. 4:**
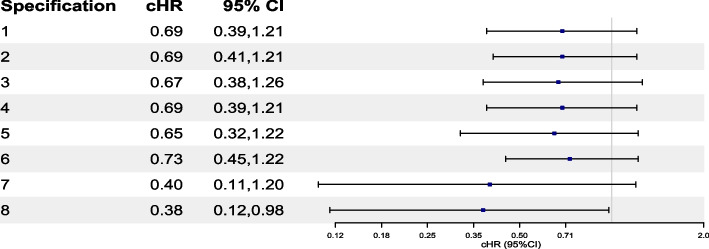
Forest plot of cHR and 95% CI for the causal effect of “always treat with brigatinib” versus “always treat with crizotinib” on OS investigated through specifications 1 to 8 using IPCW. LTFU is assumed at random; Death is censored by a minimum of treatment switching and LTFU/AC. Details about Specifications 1 (full model) and 2 (restricted model) are also available in Tables S4 and S5 in ESM 3. Refer to Table S3 in ESM 4 for a full list of variable abbreviations used in this figure. Spec 1: $\text {AGE}, \text {IDS}_{4}, \text {ICS}_{2}, \text {LI}_{4}, \text {ST}_{4}, \text {SM}_{2}, \text {FUT}, \text {ECOG}_{3}, \text {TLS}, \text {TDP}, \text {TDP}^2$. Spec 2: $\text {AGE}, \text {IDS}_{4}, \text {ST}_{4}, \text {FUT}, \text {TLS}, \text {TDP}, \text {TDP}^2$. Spec 3: Same as Spec 1 but with $\text {FUT}^S, \text {TLS}^S,\text {Age}^S,\text {TDP}^S$ instead of their linear terms. Spec 4: Same as Spec 1 but with $\text {ECOG}_{2}$ instead of $\text {ECOG}_{3}$. **Spec 5**: Same as Spec 2 but with $\text {FUT}^S$, $\text {TLS}^S$, $\text {Age}^S$, and $\text {TDP}^S$ instead of their linear terms. Spec 6: Same as Spec 2 but without $\text {IDS}_{4}$. Spec 7: Same as Spec 1 but without $\text {TDP}$ and $\text {TDP}^2$. Spec 8: Same as Spec 2 but without $\text {TDP}$ and $\text {TDP}^2$. cHRs were estimated from a weighted pooled logistic regression using weights for treatment switching and formulated based on equation (5) provided in ESM 2. Reported CIs were estimated using a non-parametric bootstrap procedure based on 1000 samples. cHR, cumulative hazard ratio; CI, confidence interval; OS, overall survival, IPCW, inverse probability of censoring weights; ESM: electronic supplementary material

In Table 5 we present the results of our analyses with IPCW that adjust for treatment switching only. In specifications 1 to 8, we report the summary statistics of stabilized weights as well as cHRs (95% CI) and RRs (95% CI) from a weighted pooled logistic regression for the difference in OS between the two treatment groups adjusted for switching. The distribution of stabilized weights was similar across specifications 1 to 6, with means ranging between 0.95 to 0.97 and SDs between 0.24 to 0.30. The reported cHRs and RRs were relatively similar across the six model specifications ranging between 0.65 (0.32,1.22) and 0.73 (0.45,1.22) for cHR and 0.72 (0.43,1.17) to 0.79 (0.54,1.17) for RR. In specifications 7 and 8, we explored the effect of removing the linear and quadratic terms for time to disease progression from specifications 1 and 2 respectively. Although we did observe a mean closer to 1, the SDs for the weights became notably larger ($> 1$) and the resulting cHR and RR dropped down to 0.40 (0.11,1.20) and 0.53 (0.32,1.16) respectively in specification 7 and 0.38 (0.12,0.98) and 0.52 (0.32,0.98) in specification 8. To explore the impact of truncating extreme weights, we progressively truncated the weights from specification 7 as detailed in Table 6. Despite the increase in precision as weights become more truncated, we observed potential bias introduced when truncating beyond the $5^\text {th}$ and $95^\text {th}$ percentiles. Analysis with the different truncated weights resulted in an RR of 0.66 (0.49,1.13), 0.81 (0.59,1.28), 0.87 (0.61,1.32) and 0.87 (0.61,1.32) with increasing levels of truncations. A similar trend was observed with cHR. Findings from Table 5 are also visualized through a forest plot in Fig. 4 and Figure S5 in ESM 7. In Table S11 in ESM 8 we report the summary statistics of unstabilized weights and corresponding effect estimates.

**Fig. 5:**
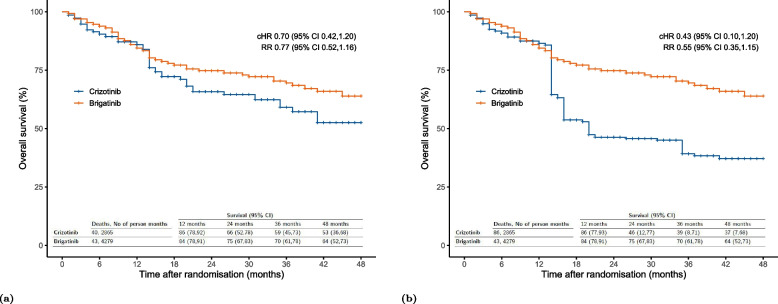
Counterfactual weighted Kaplan-Meier (KM) survival curves comparing overall survival between “always treat with brigatinib” and “always treat with crizotinib”; Survival probabilities are derived from the KM estimator, Cumulative Hazard Ratios (cHRs) and Risk Ratios (RRs) are estimated from a weighted pooled logistic regression model; cHR is formulated based on equation (5) provided in the Electronic Supplementary Material (ESM) 2; RR is formulated as the ratio of the cumulative risks by month 48; Estimates are weighted by the product of inverse probability of censoring weights for lost to follow-up/administrative censoring and switching; In panel (**a**) weight estimates are derived from specification 4 in Table 4 and specification 4 in Table 5. In panel (**b**) weight estimates are derived from specification 4 in Table 4 and specification 7 in Table 5

In our analysis that adjusts for both LFTU/AC and treatment switching, we chose specification 4 for the LTFU/AC and treatment switching models. This decision was a result of a thoughtful balance between including an adequate number of flexibly modelled confounders in the weighting model and generating well-behaved weights. Although the results from specification 1 did not differ from that of specification 4, we settled on 4 as it closely compares to the parametric g-formula analysis in terms of the number of ECOG levels. The resulting cHR and RR with their corresponding (95%C) were 0.70 (0.42,1.20) and 0.77 (0.52,1.16) respectively. This result is based on a weighted pooled logistic regression model using stabilized weights for treatment switching and LTFU. Results from a CoxPH reported a similar HR of 0.71 (0.42, 1.21). Using the KM estimator resulted in a cHR of 0.70 (0.39,1.31) and an RR of 0.76 (0.50,1.23). Figure 5a and b display the KM curves from this analysis and the sensitivity analysis that replaces specification 4 for switching adjustment with specifications 7.

#### Results from the parametric g-formula analysis

**Fig. 6:**
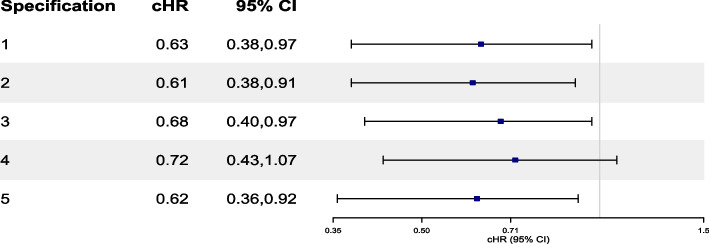
Forest plot of cHR and their 95% CI for the causal effect of “always treat with brigatinib” versus “always treat with crizotinib” on OS investigated through specifications 1 to 5 using the parametric g-formula. Details about Specifications 1, 2 and 3 are available in Tables S6, S7 and S8 in the Electronic Supplementary Material (ESM) 5. Specifications 4 and 5 are the same as Specification 1 but replacing 2 categories of ECOG with 3 categories for Specification 4 and without progression time for Specification 5. Reported CIs were calculated using a non-parametric bootstrap procedure based on 500 samples (with 60 replicate failures observed for specification 4). cHR, cumulative hazard ratio; CI, confidence interval; OS, overall survival

**Fig. 7:**
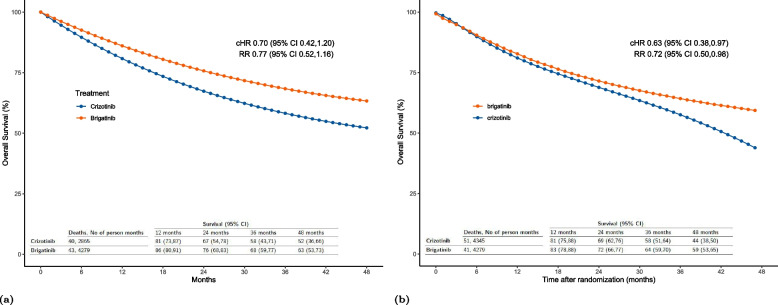
Counterfactual parametric survival curves comparing overall survival between “always treat with brigatinib” and “always treat with Crizotinib”; Cumulative Hazard Ratios (cHRs), Risk Ratios (RRs) and survival probabilities are estimated from a pooled logistic regression model; cHR is formulated based on equation (5) provided in the Electronic Supplementary Material (ESM) 2; RR is formulated as the ratio of the cumulative risks by month 48. In panel (**a**) estimates are weighted by the product of the inverse probability of censoring weights for LTFU and switching from specification 4 in Table 4 and specification 4 in Table 5. In panel (**b**) estimates are derived from the parametric g-formula from specification 1 in Table 7. Reported CIs are calculated using a non-parametric bootstrap procedure based on 1000 samples for panel (**a**) and 500 samples for panel (**b**)

In Table 7, we present the results of our analyses with the parametric g-formula. In specification 1, the estimated 48-month risk was 0.56 (0.41,0.67) for “always treat with crizotinib” and 0.41 (0.29,0.49) for “always treat with brigatinib”. The 48-month RR was 0.72 (0.50,0.98). The cHR for crizotinib versus brigatinib was 0.63 (0.38,0.97). With the exception of specification 4, which uses 3 categories of ECOG and reports a cHR of 0.72 (0.43,1.07) and an RR of 0.79 (0.54,1.05), the results did not materially change across the remaining sensitivity analyses. Findings from Table 7 are also visualized through a forest plot as shown in Fig. 6 and Figure S6 in ESM 7. The counterfactual survival curves from specification 1 and the previous IPCW analysis are shown in Fig. 7.

## Discussion

### Research problem and key findings

In the ALTA-1L trial, nearly half of the patients in the crizotinib arm switched to brigatinib after disease progression. Treatment switching (following disease progression) and LTFU/AC at each visit may have been influenced by various time-varying and baseline covariates, introducing causal inference and statistical challenges in estimating overall survival (OS) in clinical trials. Beyond its clinical relevance, OS is also a critical outcome in health technology assessments, informing the cost-effectiveness of introducing new treatments to the existing standard of care. These policy assessments often require estimates of treatment effects that reflect real-world scenarios where switching to an experimental treatment is not yet an option. Our analysis has therefore focused on estimating the effect on OS under the strategies “always treat with brigatinib” versus “always treat with crizotinib”, eliminating switching and LTFU/AC events. To achieve this, we utilized g-methods, which address the limitations of conventional regression methods that introduce selection bias and over-adjustment in the presence of treatment-confounder feedback.

The published findings from the sensitivity analysis carried out by the ALTA-1L study team, applying the IPCW approach to account for treatment switching, have shown a substantial reduction in the HR of death (HR for death: 0.50 (0.28–0.87)) as compared to the ITT results. Our first objective was to apply the IPCW method to reproduce the results from the ALTA-1L based on the information provided. While our attempts to reproduce this result were generally successful (HR: 0.51 (0.28–0.93)), we did encounter slight numerical differences. These differences may be attributed to differences in the definition of time intervals when structuring the data, differences in the approach to limit extreme weights and limitations in the level of detail available in the provided Statistical Analysis Plan (SAP). However, our re-analyses with the IPCW and parametric g-formula approaches – that additionally consider progression status and time to progression as potential confounders – have shown distinct results from the published ones, and demonstrated greater robustness across a range of sensitivity analyses. Overall, our results consistently revealed that the survival benefit of brigatinib is likely to be higher than what was observed in the ITT analysis when accounting for the effect of switching from crizotinib to brigatinib.

Our analyses with g-methods required a number of modelling choices that strongly affected our results. This complexity was compounded by our decision to report an extensive range of sensitivity analyses. Hiding the potential consequences of the different choices within this “garden of forking paths” [32] could have significant implications, especially in clinical applications. It was therefore important to us to extensively and clearly report our sensitivity analyses, and publicly share our analysis R script. In light of our application of these methods, we also foresee a rather overlooked challenge in the process of drafting an SAP following the International Council for Harmonisation of Technical Requirements for Pharmaceuticals for Human Use (ICH)-E9 guidance with a specified set of models that both set the course for an entire analysis. Different data patterns and unforeseen challenges might significantly impact the applicability of such methods where researchers might find themselves in a dilemma trading off between providing a complete, predefined analysis plan and allowing for flexibility to adapt to unanticipated complexities that may emerge later in the analysis.

### Interpretation of the IPCW results

When estimating the weights in our IPCW analyses, we considered a set of predictors that were either prognostic of survival [33] or were jointly predicting switching and survival [9]. Through our analysis, we have noticed that including variables that only affect the probability of survival (without considering switching) compared to a rather restricted model that includes variables that are jointly predictive of switching and death did not yield different results. This suggests that including predictors that affect survival only did not meaningfully improve efficiency in our estimates. Additional sensitivity analyses that either aimed to relax the linearity assumption or explore the influence of potential residual confounding within categories of the confounders have shown to be relatively consistent across the aforementioned sensitivity analysis with results.

Two additional IPCW sensitivity analyses have shown diverging results further away from the null with misbehaved weights when one single covariate (time to disease progression) was excluded from the analysis. Referring to the DAG presented in Fig. 1 and our multi-variate analysis reported in Tables S4 and S5 in ESM 3, time to progression in ($L_{1}$) has shown a notable proportional relationship with treatment intake ($A_{1}$). Upon examination of the respective individual-level data, we noticed that observations with extreme weights were those who experienced earlier disease progression. Within this subgroup of data, we identified three instances of patient deaths that were associated with disproportionately high weights behaving as leverage points. This has probably contributed to an overestimation of deaths within the control group and a perceived advantage for the experimental group on survival. Adjusting the weights of these three leverage points to the average value of the maximum weights from the first six specifications has notably shifted the cHR from their initial value of 0.40 (0.11,1.20) to 0.68 (0.38,1.49). This underscores the considerable importance of addressing extreme weights in the interpretation of our findings.

In our study, nearly half the patients (53%) in the crizotinib arm did not switch to brigatinib. These patients formed the basis of the IPCW survival estimates in the control arm. A simulation study [34–36] has shown that high levels of bias can be produced when switching proportions exceeded 85%, we therefore did not consider the size of the remaining non-switchers as a substantial source of uncertainty associated with our results. To relax the assumption of differential censoring due to LTFU, we combined the weights of switching with LTFU/AC weights in the outcome model to estimate the causal effect of our previously defined hypothetical treatment strategies on OS.

### Interpretation of parametric g-formula results

The results from our parametric g-formula analyses suggest that the treatment effects (cHRs and RRs) after 4 years are lower than the reported ITT results. Since the parametric g-formula relies on specifying a number of parametric models, we conducted several sensitivity analyses to assess the robustness of our results to different modelling decisions. We found that the estimates (e.g., counterfactual risks, RRs, cHRs) were similar across the five different analyses. Interestingly, omitting the “time to progression” variable from the outcome model did not strongly affect the g-formula estimates, contrary to our IPCW analyses.

The parametric g-formula has seen relatively limited applications compared to IPW approaches, particularly when estimating PP effects in 2-arm RCTs. One practical barrier to applying this method is its complexity to implement. In fact, the analyses conducted in this project involved a nonstandard application of the relatively new gfoRmula R package and required a number of updates to the package.

### Comparability between methods

Results from a relevant RCT (ALEX study) [37] comparing alectinib (also a next-generation ALK tyrosine kinase inhibitor) to crizotinib in ALK-positive NSCLC, however unaffected by treatment switching, have shown an HR of 0.67 (0.46–0.98), a result that is relatively close to the ones we report in our analysis. While acknowledging that the two trials have differences in patient characteristics and background treatment, we believe that the comparable findings from the ALEX study offer valuable supporting evidence that reinforces our results especially that the PFS results from the ALEX trial were relatively similar to those reported in the ALTA-1L.

### Plausibility of assumptions

The IPCW and parametric g-formula methods operate under five key assumptions. We considered consistency (where treatment and LTFU/AC variables are well-defined) and no measurement errors as plausible assumptions given the controlled experimental design of the ALTA-1L trial. The application of both IPCW and g-formula also requires the assumption that all baseline and TVCs that jointly predict the outcome and switching are observed. While it is advised to adjust for as many confounding variables as possible to satisfy this exchangeability assumption, it can be challenging to do so in small-medium-sized trials. The exchangeability assumption may be violated to some extent in our analyses, as we performed variable selection (among a set of possible confounders based on expert domain knowledge) in the nuisance function models due to the limited sample size of the trial.

To help address this, we provided the reader with a clear description of the covariates considered in our analysis and presented a series of sensitivity analyses to provide more comprehensive results. For the positivity assumption (where there is adequate variability in the crossover and LTFU patterns) we did not observe any obvious violation of positivity in most of our analyses.

All models need to be correctly specified to obtain valid estimates. While g-formula makes relatively stronger model assumptions in the sense that multiple models need to be fitted (outcome and time-varying confounding variables), we took proactive steps in this regard by adopting a series of sensitivity analyses. In regards to the correct model specification of the weighting model within the IPCW approach, we carefully examined the resulting weights in Tables 4 and 5, particularly focusing on identifying any potential extreme values. With the exception of specifications 7 and 8 from Table 5, we have noticed a rather stable behaviour without instances of extreme values. We have also noticed that addressing the large weights with a range of truncations has a bias-variance trade-off where the bias increases and the variance decreases as the weights become more truncated. Similar observations have been made in applied and methodological studies [5].

### Alternative methods

There are a number of alternative approaches to estimate PP effects each relying on different assumptions than the g-methods we used. One example we considered in this work, is the Rank Preserving Structural Failure Time Model (RPSFTM) approach proposed by Robins [13]. Unlike IPCW and parametric g-formula, this approach does not require the “no unmeasured confounding” assumption, however assumes a common treatment effect. We did not find it reasonable to assume that switchers in the control arm would attain the same benefit from treatment as those who initially received the experimental treatment at randomization.

Another approach is the Two-Stage Estimation (TSE) [26] proposed by Latimer in 2014 which assumes the absence of time-dependent confounding between the time of disease progression (taken as a secondary baseline) and the time of treatment switch. The application of the two-stage method was not considered suitable, mainly because of the time lag between disease progression and treatment switching for many patients. By using such a method, differences between switchers and non-switchers may not be adequately accounted for within this framework.

The application of an “improved TSE”, a methodology recently introduced by Latimer in 2020 [8] which uses structural nested models and g-estimation to account for time-dependent confounding seems to be a viable approach for future work.

### Open science and reproducible research

Despite our successful attempt to reproduce the published results, the various methodological options and “forks in the road” encountered through our analyses underscore a rather challenging task that researchers would probably encounter while attempting to replicate or even reproduce someone else’s work. This distinct topic is beyond the scope of our current work but calls for serious discussions on comprehensive and extensive documentation and collaborative efforts, to ensure that replicability work in complex domains like the use of causal methods in clinical trials yield reliable results.

Our efforts to demonstrate our analysis process and publicly share our analysis R script led us to create the synthetic data to overcome the limitation of sharing the real clinical trial data. For this, we generated a synthetic version of individual-level data that reflects the relationships among baseline and time-dependent covariates from the ALTA-1L over 50 discrete time points. To achieve this, we utilised estimates of baseline and TVC derived from the summary of g-formula model fits, particularly in the control arm, to inform the distribution of treatment switching, outcome, censoring and other TVC. Our methodology further incorporated deterministic information in the form of restrictions to align our simulation with the specific context of the ALTA-1L trial. Our synthetic data and shared R code should not be used to reproduce the published results or draw clinical conclusions, but should only serve as a tool for gaining insights into our analysis process.

**Table 8:** IPCW and parametric g-formula: a road map to handle treatment switching and attrition bias

1.	**Recognize the different approaches to analyzing Randomized Controlled Trials (RCTs)**
	• ITT analysis - though does not necessitate adjusting for switching - might underestimate the treatment effect of interest
	• Per-protocol analysis - though prone to selection bias - remains an important analysis in estimating the effectiveness of treatment in the decision-making process
	• Explore the potential of an appropriately conducted PP analysis for more meaningful insights into treatment effects
2.	**Define your causal estimand(s)**
	• Facilitate a collaborative discussion based on the literature, clinical experience, and relevant guidelines to define your estimand(s)
3.	**Understand the methodological challenges and opportunities in estimating your causal estimand**
	• Directed acyclic graphs are helpful tools for understanding the structural relationship between the variables of interest
	• Conventional regression methods are typically biased in the presence of treatment-confounder feedback
	• G-methods such as IPCW and the parametric g-formula are potential methods to address treatment switching
4.	**Apply inverse probability of censoring weighting**
	• IPCW censors data at switching and creates a pseudo-population in which the distribution of confounding factors is the same across the treatment regimens
	• Inspect your data for the proportion of switching^a^ and possible violations of positivity assumptions^b^
	• If applicable, incorporate deterministic knowledge of some of the variables to help mitigate bias from model misspecification
	• Document the type of the model (e.g. pooled logistic regression) that is used to calculate stabilized and non-stabilized weights
	• Inspect and clearly report the distribution of the estimated weights^c^
	• Carefully handle any truncation of weights and report the different levels of truncation
	• Apply the multiplication of different inverse probability of censoring weights in the outcome model to adjust for both switching and lost to follow-up/administrative censoring
	• Estimate the 95% CIs by bootstrapping the entire procedure (weighting and outcome estimation) to account for correlated data and the weight estimation process
	• Perform a range of sensitivity analyses to explore the potential consequences of the different model choices
5.	**Apply the parametric g-formula**
	• Parametric g-formula estimates the counterfactual risks of the treatment regimens at each time point by performing a Monte Carlo simulation based on the fitted models of exposure, outcome and confounders
	• If applicable, incorporate deterministic knowledge of some of the variables to help mitigate bias from model misspecification
	• Inspect the simulated data set to check that the intervention, covariate simulation, and restrictions (if applicable) were applied correctly
	• Inspect the estimated coefficients in the fitted models for any irregularities
	• Compare the parametric g-formula estimates of the covariate means and outcome risks under the natural course estimates to the observed (or inverse probability weighted) covariate means and risks
6.	**Comparison between IPCW and the parametric g-formula**
	• IPCW and parametric g-formula both operate under the following five different assumptions: no unmeasured confounding, positivity, consistency, correct model specification, and no measurement error
	• Compare the results from the IPCW and parametric g-formula. While large differences between the two approaches are alarming, smaller differences - though do not guarantee any absence of model misspecification - might still be reassuring
7.	**Address and report thoroughly all methodological challenges in your analyses**
	• Transparently report any divergent results from the conducted sensitivity analysis
	• Acknowledge the strengths and limitations of the IPCW and parametric g-formula methods
	• Communicate opportunities, uncertainties and sources of bias in your analysis

^a^ Proportion of switching: High switching probability might be problematic

^b^ Possible violations of positivity assumptions: Is the Probability of switching equal to 1 within certain levels of the confounder?

^c^ Extreme weights can signal model misspecification

## Conclusion

Applying an ITT approach likely underestimated the treatment effect of brigatinib versus crizotinib. While ITT analyses provide important information from randomized clinical trials, appropriately adjusted PP analyses can provide decision makers with additional information about the effectiveness of treatment. We have seen that adjustment methods that account for treatment switching have various limitations and their suitability depends on the characteristics of the trial data under evaluation. Our analyses illustrated opportunities and challenges encountered when employing methods to estimate PP effects. Overall, our results have shown how these methods could be successfully implemented in a relatively small-sized trial and how different model specifications can lead to considerably different results. To help data analysts better understand key points in applying IPCW and parametric g-formula methods in similar settings, we conclude our paper by offering a road map as shown in Table 8.

## Supplementary Information

Supplementary Material 1 provides a data dictionary for the IPCW and parametric g-formula analyses conducted on the structured synthetic data.

Supplementary Material 2 provides details about the different concepts of hazard ratio, discrete hazard ratio and cumulative hazard ratio as applied in this paper.

Supplementary Material 3 provides the parameter estimate values and odds ratios for the coefficients used to estimate the weights for the IPCW analysis.

Supplementary Material 4 provides a list of abbreviations for the covariates used in the inverse probability of censoring weights and parametric gformula methods.

Supplementary Material 5 provides the list of covariates included in the parametric g-formula analysis.

Supplementary Material 6 provides the number of replicate failures in each analysis.

Supplementary Material 7 provides forest plots of risk ratios and additional supplementary survival plots.

Supplementary Material 8 provides results from the IPCW analyses using unstabilized weights.

## Data Availability

No datasets were generated or analysed during the current study.

## Abbreviations

AC: Administrative censoring
ALK: Anaplastic lymphoma kinase
ALTA-1L: ALK in lung cancer trial of brigatinib in 1st line
BIRC: Blinded independent review committee
cHR: Cumulative hazard ratio
CI: Confidence interval
CNS: Central nervous system
CoxPH: Cox proportional hazard
DAG: Directed acyclic graph
dHR: Discrete hazard ratio
ECOG: Eastern cooperative oncology group
ESM: Electronic supplementary material
GEEs: Generalized estimating equations
HR: Hazard ratio
ICH: International council for harmonisation of technical requirements for pharmaceuticals for human use
IPCW: Inverse probability of censoring weights
IPW: Inverse probability weighting
KM: Kaplan meier
ITT: Intention to treat
LTFU: Loss to follow-up
MSM: Marginal structural model
NSCLC: Non-small cell lung cancer
OR: Odds ratio
OS: Overall survival
PFS: Progression-free survival
PP: Per-protocol
RCTs: Randomized controlled trials
RECIST: Response evaluation criteria in solid tumours
RPSFTM: Rank preserving structural failure time model
RR: Risk ratio
SAP: Statistical analysis plan
SD: Standard deviation
TSE: Two-stage estimation
TVC: Time-varying covariate
