# Supplementary material for: Addressing treatment switching in the ALTA-1L trial with g-methods: exploring the impact of model specification
Source: BMC Med Res Methodol. 2024 Dec 20;24:314. doi: 10.1186/s12874-024-02437-6 (PMC11660711; doi:10.1186/s12874-024-02437-6)
Supplement: Supplementary file 1 — Supplementary Material 1 provides a data dictionary for the IPCW and parametric g-formula analyses conducted on the structured synthetic data. [file 12874_2024_2437_MOESM1_ESM.pdf]

Addressing Treatment Switching Bias with G-methods: Exploring the Impact of Model Specification

Amani Al Tawil*[1,2], Sean McGrath[3], Robin Ristl†[4], and Ulrich Mansmann†[1,2]

[1] *Institute for Medical Information Processing, Biometry, and Epidemiology (IBE), Faculty of Medicine, Ludwig-Maximilians-Universität München*
[2] *Pettenkofer School of Public Health, Faculty of Medicine, Ludwig-Maximilians-Universität München*
[3] *Department of Biostatistics, Harvard T.H. Chan School of Public Health*
[4] *Center for Medical Data Science, Medical University of Vienna*

Electronic Supplementary Material 1

Data Dictionary

*Correspondence: altawil@ibe.med.uni-muenchen.de
†Equally contributed

Data Dictionary

**Participant Identifier**	**Variable Name**	**Variable Type**	**Coding**
Participant ID	id	Numerical	Unique numerical number

**Time**	**Variable Name**	**Variable Type**	**Coding**
Follow-up time	time	Discrete, numerical	Follow-up time (months since baseline)
Progression time	progtime	Continuous	Time at progression (months since baseline)

**Baseline Covariates**	**Variable Name**	**Variable Type**	**Coding**
Age	AGE	Continuous	Years
Sex	SEX	Binary	0: Male<br>1: Female
Race	RACEGR1	Binary	0: Non-asian<br>1: Asian
Eastern Cooperative Oncology Group score (ECOG)	ECOGGR1	Binary	0: 0<br>1: 1,2,3,4 or 5
Smoking history	SMKHISGR1	Binary	0: Never<br>1: Ever
Measurable intracranial central nervous system (CNS) disease	MICNSFL	Binary	0: No measurable intracranial disease<br>1: Measurable intracranial disease
Prior radiation therapy	PRRADYN	Binary	0: No prior radiation therapy<br>1: Prior radiation therapy
Strata at randomization (baseline brain metastases and previous chemotherapy)	STRATRAN	Categorical	0: No iCNS metastasis/No prior chemo<br>1: iCNS metastasis/No prior chemo<br>2: No iCNS metastasis/Prior chemo<br>3: iCNS metastasis/Prior chemo
Initial cancer diagnosis stage	DIAGINITGR3	Categorical	0: IV<br>1: IIIB<br>2: IIIA<br>3: IIA,IB or IA
Lung involvement at study entry	LIASE	Categorical	0: Both<br>1: Right<br>2: Left<br>3: Lung not involved

**Time-Varying Covariates**	**Variable Name**	**Variable Type**	**Coding**
Disease progression	progtd	Binary	0: No progression<br>1: Progression
Intracranial disease progression	icprogtd	Binary	0: No intracranial progression<br>1: Intracranial progression
ECOG	ecogtdGR1	Binary	0: 0<br>1: 1,2,3,4 and 5
	ecogtdGR2	Categorical	0: 0<br>1: 1<br>2: 2,3,4 or 5
Target-lesion size	tltd_wins	Continuous	mm
Switching status	xotd	Binary	0: No switching<br>1: switching

**Treatment**	**Variable Name**	**Variable Type**	**Coding**
Treatment randomized	plannedtrt	Binary	0: Crizotinib<br>1: Brigatinib
Treatment received	trttd	Binary	0: Crizotinib<br>1: Brigatinib

**Outcome**	**Variable Name**	**Variable Type**	**Coding**
Death	deathtd	Binary	0: Alive<br>1: Died

**Lost to follow-up**	**Variable Name**	**Variable Type**	**Coding**
Censoring due to loss to follow-up / administrative censoring	censoringtd_new	Binary	0: No censoring<br>1: Censoring
