# Supplementary material for: Addressing treatment switching in the ALTA-1L trial with g-methods: exploring the impact of model specification
Source: BMC Med Res Methodol. 2024 Dec 20;24:314. doi: 10.1186/s12874-024-02437-6 (PMC11660711; doi:10.1186/s12874-024-02437-6)
Supplement: Supplementary file 2 — Supplementary Material 2 provides details about the different concepts of hazard ratio, discrete hazard ratio and cumulative hazard ratio as applied in this paper. [file 12874_2024_2437_MOESM2_ESM.pdf]

Addressing Treatment Switching Bias with G-methods: Exploring the Impact of Model Specification

Amani Al Tawil*[1,2], Sean McGrath[3], Robin Ristl†[4], and Ulrich Mansmann†[1,2]

[1] *Institute for Medical Information Processing, Biometry, and Epidemiology (IBE), Faculty of Medicine, Ludwig-Maximilians-Universität München*
[2] *Pettenkofer School of Public Health, Faculty of Medicine, Ludwig-Maximilians-Universität München*
[3] *Department of Biostatistics, Harvard T.H. Chan School of Public Health*
[4] *Center for Medical Data Science, Medical University of Vienna*

Electronic Supplementary Material 2

Hazard

*Correspondence: altawil@ibe.med.uni-muenchen.de
†Equally contributed

Hazard

The following will specify the concepts of hazard ratio (HR), discrete hazard ratio (dHR) and cumulative hazard ratio (cHR) as used in this paper.

The hazard at time t of an event time T given covariates x is defined by Equation (1).

$$\lim_{\Delta \to 0} \frac{P(T \in [t, t+\Delta] | T > t, x)}{\Delta} = \lambda_x(t) \tag{1}$$

In case of a Proportional Hazards (PH) model it holds $\lambda_x(t) = \lambda_0(t) \cdot \mathrm{HR}(x)$. Here, $\lambda_0(t)$ is the baseline hazards function and $\mathrm{HR}(x)$ is a specific factor changing the hazard to a group of subjects with specific covariates x (e.g., indicator of assignment to experimental versus control group). In the PH model, it is assumed that $\lambda_0(t)$ holds for a well-defined baseline group (where all components of x are set to 0) and $\mathrm{HR}(x)$ quantifies the change in hazard between a group of subjects defined by x and the baseline group.

Several of the approaches we apply model the expression $P(T \in [t, t+\Delta] \mid T > t, x)$ for small but fixed Δ by a logistic regression as shown in Equation (2).

$$P(T \in [t, t+\Delta] \mid T > t, x) = \frac{e^{LP(t,x)}}{1 + e^{LP(t,x)}} \tag{2}$$

Here the linear predictor $LP(t, x)$ depends on the time t and the covariates x (Equation (3)).

$$LP(t, x) = a_0 + a_1 \cdot t + a_2 \cdot t^2 + \sum_{i=1}^{k} b_i \cdot x_i \tag{3}$$

Given the small time interval $[t, t + \Delta]$ the probability that the event may happen is assumed to be quite small (Equation (4)),

$$P(T \in [t, t+\Delta] \mid T > t, x) \sim e^{LP(t,x)} = f(t) \cdot g(x) \tag{4}$$

The term g(x) is called discrete hazard ratio (dHR) which is the product of odds ratios defined by the vectors b and x. It approximates the HR in case the proportional hazards assumption holds.

The cumulative hazard ratio (cHR) between two groups (defined by x and y) at time t is defined by Equation (5):

$$cHR_{t,x,y} = \frac{\log(P(T > t \mid x))}{\log(P(T > t \mid y))} \tag{5}$$

The expression is motivated by the structure of the PH model which implies (Equation (6))

$$P(T > t \mid x) = \exp\left(-HR(x)\int_0^t \lambda_0(s)\,ds\right) \tag{6}$$

In case of a PH model the $cHR_{t,x,y} = HR(x-y)$. It also holds that $dHR_{t,x,y} = g(x-y)$ which approximates the HR in case the PH assumption is fulfilled.
