# Supplementary material for: Addressing treatment switching in the ALTA-1L trial with g-methods: exploring the impact of model specification
Source: BMC Med Res Methodol. 2024 Dec 20;24:314. doi: 10.1186/s12874-024-02437-6 (PMC11660711; doi:10.1186/s12874-024-02437-6)
Supplement: Supplementary file 3 — Supplementary Material 3 provides the parameter estimate values and odds ratios for the coefficients used to estimate the weights for the IPCW analysis. [file 12874_2024_2437_MOESM3_ESM.pdf]

Addressing Treatment Switching Bias with G-methods: Exploring the Impact of Model Specification

Amani Al Tawil[*1,2], Sean McGrath[3], Robin Ristl[†4], and Ulrich Mansmann[†1,2]

[1] *Institute for Medical Information Processing, Biometry, and Epidemiology (IBE), Faculty of Medicine, Ludwig-Maximilians-Universität München*

[2] *Pettenkofer School of Public Health, Faculty of Medicine, Ludwig-Maximilians-Universität München*

[3] *Department of Biostatistics, Harvard T.H. Chan School of Public Health*

[4] *Center for Medical Data Science, Medical University of Vienna*

Electronic Supplementary Material 3

Parameter Estimate Values and Odds Ratios for the Coefficients of IPCW Method

[*] Correspondence: altawil@ibe.med.uni-muenchen.de

[†] Equally contributed

Table S1: Pooled logistic regression analysis on the probability of being censored defined as lost to follow-up/administrative censoring, conditional on baseline and TVCs, to calculate the denominator of the weights - Full model

**Specification 1**★		**Loss to follow-up**			
Variable		**Control arm**		**Experimental arm**	
		**Estimate (SE)**	**OR (95% CI)**†	**Estimate (SE)**	**OR (95% CI)**†
Intercept		1.505 (2.887)		-7.957 (2.951)	
Age in yrs (continuous)		-0.301 (0.112)	0.74 (0.595,0.921)	0.136 (0.107)	1.145 (0.928,1.414)
Age in yrs (quadratic)		0.003 (0.001)	1.003 (1.001,1.005)	-0.001 (0.001)	0.999 (0.997,1.001)
Female Sex		NA	NA	-0.426 (0.317)	0.653 (0.351,1.216)
Race	Non-Asian	NA	NA	Reference	Reference
	Asian	NA	NA	0.297 (0.351)	1.346 (0.676,2.679)
Initial diagnosis stage	IV	Reference	Reference	Reference	Reference
	IIIB	-0.045 (0.521)	0.956 (0.344,2.653)	0.78 (0.54)	2.181 (0.757,6.283)
	IIIA	1.377 (0.675)	3.961 (1.055,14.873)	-0.485 (0.851)	0.615 (0.116,3.262)
	IA,IB,IIA	0.524 (0.551)	1.688 (0.573,4.971)	0.297 (0.69)	1.346 (0.348,5.201)
Measurable intracranial CNS disease		0.132 (0.828)	1.141 (0.225,5.782)	0.134 (0.559)	1.143 (0.382,3.418)
Lung involvement at study entry	Both	Reference	Reference	Reference	Reference
	Right	-0.062 (0.365)	0.939 (0.46,1.92)	-0.205 (0.352)	0.815 (0.409,1.623)
	Left	-0.16 (0.382)	0.852 (0.403,1.801)	-0.604 (0.423)	0.547 (0.239,1.252)
	Lung not involved	-0.546 (0.714)	0.579 (0.143,2.345)	-0.353 (0.58)	0.703 (0.226,2.189)
Baseline ECOG	0	NA	NA	Reference	Reference
	1 or 2	NA	NA	-0.527 (0.395)	0.59 (0.272,1.282)
Strata at randomization	No iCNS Metastasis at baseline/No prior chemotherapy	Reference	Reference	Reference	Reference
	iCNS Metastasis at baseline/No prior chemotherapy	-0.184 (0.777)	0.832 (0.182,3.813)	-0.428 (0.518)	0.651 (0.236,1.8)
	No iCNS Metastasis at baseline/Prior chemotherapy	-0.913 (0.535)	0.401 (0.14,1.146)	-0.224 (0.418)	0.799 (0.353,1.813)
	iCNS Metastasis at baseline/Prior chemotherapy	-1.002 (0.789)	0.367 (0.078,1.723)	-0.44 (0.711)	0.644 (0.16,2.592)
History of tobacco use	Never smoked	Reference	Reference	Reference	Reference
	Ever Smoked	0.223 (0.279)	1.25 (0.724,2.159)	-0.837 (0.345)	0.433 (0.22,0.851)
Previous radiation therapy		NA	NA	0.378 (0.667)	1.46 (0.395,5.399)
Time in month (continuous)		-0.21 (0.046)	0.81 (0.741,0.886)	-0.354 (0.046)	0.702 (0.641,0.769)
Time in month (quadratic)		0.007 (0.001)	1.007 (1.006,1.009)	0.01 (0.001)	1.01 (1.008,1.012)
ECOG performance-status score at current visit	0	Reference	Reference	Reference	Reference
	1	0.284 (0.301)	1.329 (0.736,2.398)	1.232 (0.37)	3.427 (1.659,7.078)
	2+	-0.509 (0.963)	0.601 (0.091,3.964)	-0.235 (1.186)	0.79 (0.077,8.076)
Tumour lesion size at current visit		0.012 (0.005)	1.012 (1.001,1.023)	0.004 (0.006)	1.004 (0.993,1.016)
Intracranial progression at current visit		NA	NA	-0.794 (0.443)	0.452 (0.19,1.076)
Disease progression at current visit		0.455 (0.451)	1.576 (0.651,3.818)	1.398 (0.339)	4.046 (2.081,7.863)
Treatment at current visit		0.032 (0.414)	1.032 (0.459,2.323)	NA	NA

† Confidence Intervals (CIs) estimated using "sandwich" variance estimator

★ Specification 3: Same as specification 1, but replace the linear terms of time, target lesion size and age with 5 knots splines
Specification 4: Same as specification 1, but replace the step function (3 categories) for time-varying ECOG with 2 categories.

Table S2: Pooled logistic regression analysis on the probability of being censored defined as lost to follow-up/administrative censoring, conditional on baseline and TVCs, to calculate the denominator of the weights - Restricted model

**Specification 2★**		**Loss to follow-up**			
Variable		**Control arm**		**Experimental arm**	
		**Estimate (SE)**	**OR (95% CI)†**	**Estimate (SE)**	**OR (95% CI)†**
Intercept		1.47 (2.901)		-8.286 (2.526)	
Age in yrs (continuous)		-0.29 (0.107)	0.748 (0.606,0.924)	0.152 (0.092)	1.165 (0.973,1.394)
Age in yrs (quadratic)		0.003 (0.001)	1.003 (1.001,1.005)	-0.001 (0.001)	0.999 (0.997,1)
Female Sex		NA	NA	-0.434 (0.293)	0.648 (0.365,1.15)
Initial diagnosis stage	IV	Reference	Reference	Reference	Reference
	IIIB	0.065 (0.455)	1.067 (0.438,2.603)	0.788 (0.433)	2.199 (0.941,5.137)
	IIIA	1.252 (0.617)	3.498 (1.044,11.715)	-0.093 (0.493)	0.911 (0.346,2.396)
	IA,IB,IIA	0.352 (0.492)	1.422 (0.542,3.725)	0.285 (0.619)	1.33 (0.395,4.479)
Baseline ECOG	0	NA	NA	Reference	Reference
	1 or 2	NA	NA	-0.482 (0.328)	0.618 (0.325,1.175)
Strata at randomization	No iCNS Metastasis at baseline/No prior chemotherapy	Reference	Reference	NA	NA
	iCNS Metastasis at baseline/No prior chemotherapy	-0.247 (0.427)	0.781 (0.338,1.802)	NA	NA
	No iCNS Metastasis at baseline/Prior chemotherapy	-1.008 (0.491)	0.365 (0.139,0.956)	NA	NA
	iCNS Metastasis at baseline/Prior chemotherapy	-0.826 (0.679)	0.438 (0.116,1.659)	NA	NA
History of tobacco use	Never smoked	NA	NA	Reference	Reference
	Ever Smoked	NA	NA	-0.897 (0.323)	0.408 (0.216,0.769)
Time in month (continuous)		-0.212 (0.045)	0.809 (0.741,0.884)	-0.36 (0.046)	0.698 (0.638,0.763)
Time in month (quadratic)		0.007 (0.001)	1.007 (1.006,1.009)	0.01 (0.001)	1.01 (1.008,1.012)
ECOG performance-status score at current visit	0	NA	NA	Reference	Reference
	1	NA	NA	1.187 (0.339)	3.278 (1.685,6.376)
	2+	NA	NA	-0.353 (1.004)	0.703 (0.098,5.03)
Tumour lesion size at current visit		0.012 (0.005)	1.012 (1.003,1.022)	NA	NA
Intracranial progression at current visit		NA	NA	-0.859 (0.394)	0.423 (0.195,0.917)
Disease progression at current visit		0.52 (0.33)	1.682 (0.881,3.211)	1.394 (0.329)	4.03 (2.115,7.677)

† Confidence Intervals (CIs) estimated using "sandwich" variance estimator

★ Specification 5: Same as specification 2, but replace the linear terms of time, target lesion size and age with 5 knots splines.
Specification 6: Same as specification 2, but remove baseline covariates: sex, baseline ECOG and initial diagnosis stage in calculating the denominator of the experimental arm

Table S4: Pooled logistic regression analysis on the probability of being censored defined as switching, conditional on baseline and time-varying covariates, to calculate the denominator of the weights - Full model

**Specification 1★**		**Treatment switching**	
Variable		**Control arm at and after progression**	
		**Estimate (SE)**	**OR (95% CI)†**
Intercept		-1.355 (1.116)	
Age in yrs (continuous)		-0.027 (0.014)	0.973 (0.946,1.001)
Initial diagnosis stage	IV	Reference	Reference
	IIIB	-0.878 (0.574)	0.416 (0.135,1.280)
	IIIA	-0.373 (0.771)	0.688 (0.152,3.122)
	IA,IB,IIA	-0.43 (0.796)	0.65 (0.137,3.094)
Measurable intracranial CNS disease		-0.312 (0.555)	0.732 (0.247,2.171)
Lung involvement at study entry	Both	Reference	Reference
	Right	0.195 (0.486)	1.216 (0.469,3.149)
	Left	0.418 (0.446)	1.519 (0.634,3.643)
	Lung not involved	0.088 (0.847)	1.092 (0.208,5.748)
Strata at randomization	No iCNS Metastasis at baseline/No prior chemotherapy	Reference	Reference
	iCNS Metastasis at baseline/No prior chemotherapy	0.009 (0.563)	1.009 (0.334,3.043)
	No iCNS Metastasis at baseline/Prior chemotherapy	1.23 (0.498)	3.42 (1.29,9.071)
	iCNS Metastasis at baseline/Prior chemotherapy	0.436 (0.621)	1.546 (0.458,5.22)
History of tobacco use	Never smoked	Reference	Reference
	Ever Smoked	-0.069 (0.333)	0.933 (0.486,1.791)
Time in month (continuous)		-0.277 (0.039)	0.758 (0.702,0.819)
ECOG performance-status score at current visit	0	Reference	Reference
	1	-0.156 (0.33)	0.855 (0.448,1.632)
	2+	-1.23 (1.01)	0.292 (0.04,2.117)
Tumour lesion size at current visit		0.008 (0.006)	1.008 (0.997,1.02)
Time to disease progression (continuous)		0.472 (0.089)	1.604 (1.348,1.908)
Time to disease progression (quadratic)		-0.006 (0.003)	0.994 (0.989,1)

† Confidence Intervals (CIs) estimated using "sandwich" variance estimator

★ Specification 3: Same as specification 1, but with 5 knots splines for time, time to disease progression, target lesion size and baseline age
Specification 4: Same as specification 1, but replace the step function (3 categories) for time-varying ECOG with 2 categories
Specification 7: Same as specification 1, but without linear and quadratic terms for time to disease progression.

Table S5: Pooled logistic regression analysis on the probability of being censored defined as switching, conditional on baseline and TVCs, to calculate the denominator of the weights - Restricted model

**Specification 2★**		**Treatment switching**	
Variable		**Control arm at and after progression**	
		**Estimate (SE)**	**OR (95% CI)†**
Intercept		-1.465 (0.928)	
Age in yrs (continuous)		-0.025 (0.013)	0.976 (0.952,1)
Initial diagnosis stage	IV	Reference	Reference
	IIIB	-0.902 (0.593)	0.406 (0.127,1.297)
	IIIA	-0.543 (0.703)	0.581 (0.146,2.305)
	IA,IB,IIA	-0.411 (0.704)	0.663 (0.167,2.632)
Strata at randomization	No iCNS Metastasis at baseline/No prior chemotherapy	Reference	Reference
	iCNS Metastasis at baseline/No prior chemotherapy	-0.139 (0.436)	0.87 (0.37,2.048)
	No iCNS Metastasis at baseline/Prior chemotherapy	1.165 (0.444)	3.205 (1.342,7.653)
	iCNS Metastasis at baseline/Prior chemotherapy	0.218 (0.591)	1.243 (0.39,3.959)
Time in month (continuous)		-0.284 (0.036)	0.752 (0.701,0.808)
Tumour lesion size at current visit		0.008 (0.005)	1.008 (0.999,1.018)
Time to disease progression (continuous)		0.486 (0.09)	1.625 (1.362,1.94)
Time to disease progression (quadratic)		-0.006 (0.003)	0.995 (0.989,1)

† Confidence Intervals (CIs) estimated using "sandwich" variance estimator

★ Specification 5: Same as specification 2, but with 5 knots splines for time, time to disease progression, target lesion size and baseline age
Specification 6: Same as specification 2, but without step function (4 categories) for initial diagnosis stage
Specification 8: Same as specification 2, but without linear and quadratic terms for time to disease progression
