# Supplementary material for: Addressing treatment switching in the ALTA-1L trial with g-methods: exploring the impact of model specification
Source: BMC Med Res Methodol. 2024 Dec 20;24:314. doi: 10.1186/s12874-024-02437-6 (PMC11660711; doi:10.1186/s12874-024-02437-6)
Supplement: Supplementary file 4 — Supplementary Material 4 provides a list of abbreviations for the covariates used in the inverse probability of censoring weights and parametric gformula methods. [file 12874_2024_2437_MOESM4_ESM.pdf]

Addressing Treatment Switching Bias with G-methods: Exploring the Impact of Model Specification

Amani Al Tawil*[1,2], Sean McGrath[3], Robin Ristl†[4], and Ulrich Mansmann†[1,2]

[1] *Institute for Medical Information Processing, Biometry, and Epidemiology (IBE), Faculty of Medicine, Ludwig-Maximilians-Universität München*
[2] *Pettenkofer School of Public Health, Faculty of Medicine, Ludwig-Maximilians-Universität München*
[3] *Department of Biostatistics, Harvard T.H. Chan School of Public Health*
[4] *Center for Medical Data Science, Medical University of Vienna*

Electronic Supplementary Material 4

Covariates used in the inverse probability of censoring weights and parametric gformula methods

*Correspondence: altawil@ibe.med.uni-muenchen.de
†Equally contributed

Tables

Table S3: Covariates used in the inverse probability of censoring weights and parametric gformula methods

	Covariates	Abbreviations
**Baseline covariates**	Age	$\text{AGE}, \text{AGE}^2, \text{AGE}^S$
	Sex	SEX_2
	Race	RACE_2
	Initial diagnosis stage	IDS_4
	Measurable intracranial Central Nervous System disease	ICS_2
	Lung involvement at study entry	LI_4
	ECOG score	ECOG_2
	Strata at randomization*	ST_4
	Smoking history	SM_2
	Prior radiation therapy	RT_2
**Time-varying covariates**	Follow-up time	$\text{FUT}, \text{FUT}^2, \text{FUT}^S$
	ECOG score	$\text{ECOG}_2, \text{ECOG}_3$
	Target-lesion size	$\text{TLS}, \text{TLS}^2, \text{TLS}^S$
	Intracranial disease progression	IDP_2
	Disease progression	DP_2
	Time to disease progression	$\text{TDP}, \text{TDP}^2, \text{TDP}^S$
	Treatment	A_2

Numerical Variables are labeled as variablename^n, where n indicates the type of transformation applied:
1: linear term, 2: quadratic term, S: spline transformation.
Categorical Variables are labeled as variablename$_n$, where n indicates the number of categories.
*Strata at randomization include baseline brain metastases and previous chemotherapy
