# Supplementary material for: Addressing treatment switching in the ALTA-1L trial with g-methods: exploring the impact of model specification
Source: BMC Med Res Methodol. 2024 Dec 20;24:314. doi: 10.1186/s12874-024-02437-6 (PMC11660711; doi:10.1186/s12874-024-02437-6)
Supplement: Supplementary file 5 — Supplementary Material 5 provides the list of covariates included in the parametric g-formula analysis. [file 12874_2024_2437_MOESM5_ESM.pdf]

Addressing Treatment Switching Bias with G-methods: Exploring the Impact of Model Specification

Amani Al Tawil*[1,2], Sean McGrath[3], Robin Ristl†[4], and Ulrich Mansmann†[1,2]

[1] *Institute for Medical Information Processing, Biometry, and Epidemiology (IBE), Faculty of Medicine, Ludwig-Maximilians-Universität München*
[2] *Pettenkofer School of Public Health, Faculty of Medicine, Ludwig-Maximilians-Universität München*
[3] *Department of Biostatistics, Harvard T.H. Chan School of Public Health*
[4] *Center for Medical Data Science, Medical University of Vienna*

Electronic Supplementary Material 5

List of Covariates Included in the Parametric G-formula Analysis

*Correspondence: altawil@ibe.med.uni-muenchen.de
†Equally contributed

List of Covariates Included in the Parametric G-formula Analysis

Table S6: Description of the covariates included in specification 1 of the parametric g-formula analysis

**Specification**†		**Control**	**Experimental**
	**Dependent Variable**	**Independent Variables**	**Independent Variables**
	Progression	time, quadratic time, race, baseline ECOG, smoking history, intracranial progression (lagged), target lesion size (lagged)	time, sex, race, baseline ECOG, smoking history, strata at randomization, intracranial progression (lagged), target lesion size (lagged)
	ECOG	time, age, quadratic age, sex, race, baseline ECOG, smoking history, measurable intracranial CNS disease, prior radiation therapy, strata at randomization, progression (lagged), progression, intracranial progression (lagged), target lesion size (lagged), treatment(lagged)	time, quadratic time, age, quadratic age, sex, race, baseline ECOG, smoking history, measurable intracranial CNS disease, prior radiation therapy, strata at randomization, intracranial progression (lagged), target lesion size (lagged)
**1**	Intracranial progression	time, age, race, prior radiation therapy, strata at randomization, progression (lagged), progression, target lesion size (lagged), treatment(lagged)	time, quadratic time, baseline ECOG, strata at randomization, progression (lagged), progression, target lesion size (lagged)
	Target lesion size	time, quadratic time, age, sex, race, baseline ECOG, measurable intracranial central nervous system(CNS) disease, prior radiation therapy, strata at randomization, progression (lagged), progression, ECOG(lagged), ECOG, intracranial progression (lagged), intracranial progression, treatment(lagged)	time, quadratic time, age, quadratic age, sex, race, baseline ECOG, strata at randomization, progression (lagged), progression, ECOG(lagged), ECOG, intracranial progression (lagged), intracranial progression
	Death	time, quadratic time, age, quadratic age, smoking history, strata at randomization, ECOG(lagged), ECOG, treatment (lagged), treatment, progression time	time, sex, measurable intracranial CNS disease, strata at randomization, ECOG(lagged), ECOG, intracranial progression (lagged), intracranial progression, progression time

Abbreviations: ECOG, Eastern Cooperative Oncology Group score; CNS, Central Nervous System

† Specification 4 from g-formula analysis is the same as specification 1 but replacing 2 categories of ECOG with 3 categories.
Specification 5 from g-formula analysis is the same as specification 1 but without progression time.

Table S7: Description of the covariates included in specification 2 of the parametric g-formula analysis

Specification†		Control	Experimental
	**Dependent Variable**	**Independent Variables**	**Independent Variables**
	Progression	time, race, baseline ECOG, intracranial progression (lagged), target lesion size (lagged)	time, race, baseline ECOG, smoking history, strata at randomization, intracranial progression (lagged), target lesion size (lagged)
	ECOG	time, age, quadratic age, race, baseline ECOG, smoking history, measurable intracranial CNS disease, prior radiation therapy, strata at randomization, progression (lagged), intracranial progression (lagged), target lesion size (lagged), treatment(lagged)	time, quadratic time, age, quadratic age, sex, race, baseline ECOG, smoking history, measurable intracranial CNS disease, prior radiation therapy, strata at randomization, intracranial progression (lagged), target lesion size (lagged)
**2**	Intracranial progression	time, age, race, prior radiation therapy, strata at randomization, progression (lagged), progression	time, baseline ECOG, strata at randomization, progression (lagged), progression
	Target lesion size	time, quadratic time, age, sex, race, baseline ECOG, measurable intracranial central nervous system(CNS) disease, prior radiation therapy, strata at randomization, progression, treatment(lagged)	time, quadratic time, age, quadratic age, sex, race, baseline ECOG, strata at randomization, progression
	Death	time, quadratic time, age, quadratic age, smoking history, strata at randomization, treatment (lagged), treatment, progression time	time, sex, measurable intracranial CNS disease, strata at randomization, intracranial progression (lagged), intracranial progression, progression time

Abbreviations: ECOG, Eastern Cooperative Oncology Group score; CNS, Central Nervous System

Table S8: Description of the covariates included in specification 3 of the parametric g-formula analysis

**Specification**[†]		**Control**	**Experimental**
	**Dependent Variable**	**Independent Variables**	**Independent Variables**
	Progression	time, race, baseline ECOG, intracranial progression (lagged), target lesion size (lagged)	time, race, baseline ECOG, strata at randomization, intracranial progression (lagged), target lesion size (lagged)
	ECOG	time, age, quadratic age, race, baseline ECOG, smoking history, measurable intracranial CNS disease, prior radiation therapy, strata at randomization, progression (lagged), intracranial progression (lagged), target lesion size (lagged), treatment(lagged)	time, age, quadratic age, sex, race, baseline ECOG, smoking history, measurable intracranial CNS disease, prior radiation therapy, strata at randomization, intracranial progression (lagged)
**3**	Intracranial progression	time, age, prior radiation therapy, strata at randomization, progression (lagged), progression	time, baseline ECOG, strata at randomization, progression (lagged), progression
	Target lesion size	time, quadratic time, age, sex, race, baseline ECOG, measurable intracranial central nervous system(CNS) disease, prior radiation therapy, strata at randomization, progression, treatment(lagged)	time, quadratic time, age, quadratic age, sex, race, baseline ECOG, strata at randomization, progression
	Death	time, age, quadratic age, strata at randomization, treatment (lagged), treatment, progression time	time, intracranial progression (lagged), intracranial progression, progression time

Abbreviations: ECOG, Eastern Cooperative Oncology Group score; CNS, Central Nervous System
