# Supplementary material for: Addressing treatment switching in the ALTA-1L trial with g-methods: exploring the impact of model specification
Source: BMC Med Res Methodol. 2024 Dec 20;24:314. doi: 10.1186/s12874-024-02437-6 (PMC11660711; doi:10.1186/s12874-024-02437-6)
Supplement: Supplementary file 6 — Supplementary Material 6 provides the number of replicate failures in each analysis. [file 12874_2024_2437_MOESM6_ESM.pdf]

Addressing Treatment Switching Bias with G-methods: Exploring the Impact of Model Specification

Amani Al Tawil*[1,2], Sean McGrath[3], Robin Ristl†[4], and Ulrich Mansmann†[1,2]

[1]*Institute for Medical Information Processing, Biometry, and Epidemiology (IBE), Faculty of Medicine, Ludwig-Maximilians-Universität München*
[2]*Pettenkofer School of Public Health, Faculty of Medicine, Ludwig-Maximilians-Universität München*
[3]*Department of Biostatistics, Harvard T.H. Chan School of Public Health*
[4]*Center for Medical Data Science, Medical University of Vienna*

Electronic Supplementary Material 6

Number (%) of bootstrap replicate failures

*Correspondence: altawil@ibe.med.uni-muenchen.de
†Equally contributed

Table S9: Number (%) of bootstrap replicate failures

	Approach	Number (%) of bootstrap replicate failures		
		**CoxPH**	**Pooled Logistic Regression**	**Kaplan-Meier**
**Intention to Treat**	Unadjusted for baseline covariates	12 (1.2)	12 (1.2)	12 (1.2)
	Adjusted for strata at randomization*	0	0	NA
	Adjusted for baseline covariates*	0	0	NA
	Marginal effect adjusted for baseline covariates	NE	0	NA
**Per Protocol**	Excluding switchers	128 (12.8)	128 (12.8)	128 (12.8)
	Censoring at switching	157 (15.7)	157 (15.7)	157 (15.7)
	Inverse probability of censoring weights*	14 (1.4)	14 (1.4)	14 (1.4)
	Parametric g-formula[✣]	NE	2 (0.4)	NE

Abbreviations: CoxPH, cox proportional hazard; CI, confidence interval; NE, not estimated; NA, not applicable
***Strata at randomization**: presence or absence of baseline brain metastases and completion of at least one full cycle of chemotherapy for locally advanced or metastatic disease (yes or no)
***Baseline covariates**: age, ECOG score, measurable intracranial CNS disease, race, sex, smoking history, strata at randomization, initial diagnosis stage, lung involvement at study entry and prior radiation therapy
***Inverse probability of censoring weight**: Estimates for the IPCW approach were estimated using a weighted pooled logistic regression model using the product of the two weights for LTFU/AC (specifications 4 in Table 4) and switching (specifications 4 in Table 5).
