# Supplementary material for: Addressing treatment switching in the ALTA-1L trial with g-methods: exploring the impact of model specification
Source: BMC Med Res Methodol. 2024 Dec 20;24:314. doi: 10.1186/s12874-024-02437-6 (PMC11660711; doi:10.1186/s12874-024-02437-6)
Supplement: Supplementary file 7 — Supplementary Material 7 provides forest plots of risk ratios and additional supplementary survival plots. [file 12874_2024_2437_MOESM7_ESM.pdf]

Addressing Treatment Switching Bias with G-methods: Exploring the Impact of Model Specification

Amani Al Tawil*[1,2], Sean McGrath[3], Robin Ristl†[4], and Ulrich Mansmann†[1,2]

[1] *Institute for Medical Information Processing, Biometry, and Epidemiology (IBE), Faculty of Medicine, Ludwig-Maximilians-Universität München*
[2] *Pettenkofer School of Public Health, Faculty of Medicine, Ludwig-Maximilians-Universität München*
[3] *Department of Biostatistics, Harvard T.H. Chan School of Public Health*
[4] *Center for Medical Data Science, Medical University of Vienna*

Electronic Supplementary Material 7

Survival and Forest Plots

*Correspondence: altawil@ibe.med.uni-muenchen.de
†Equally contributed

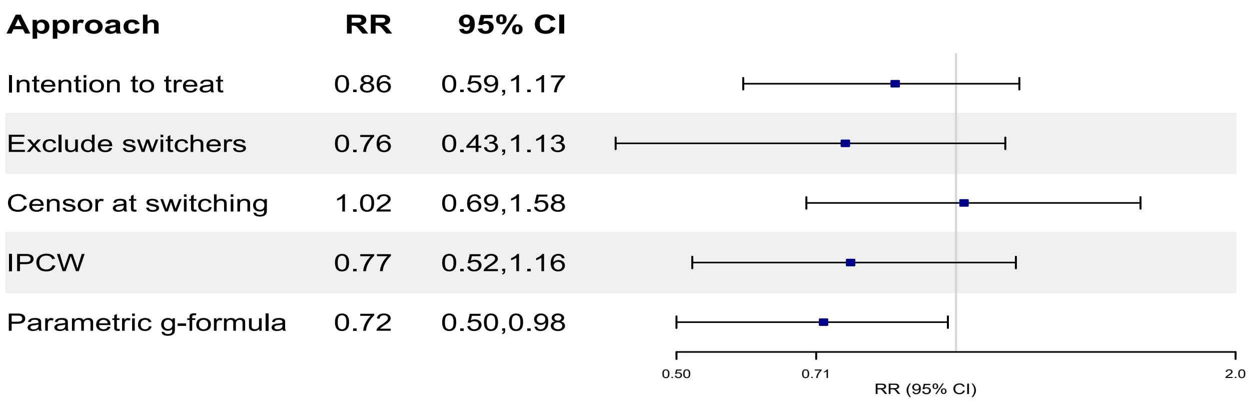

Figure S1: Forest plot of RR and their 95% CI for the effect of brigatinib versus crizotinib on OS investigated through various modelling approaches. RRs for the ITT, exclude switchers and censor at switching were estimated from a pooled logistic regression model. RR for the IPCW approach was estimated using a weighted pooled logistic regression model using the product of the two weights for LTFU/AC (specifications 4 in Table 4) and switching (specifications 4 in Table 5). RR from the parametric g-formula were estimated from specification 1 in Table 7. 95% CIs were estimated using a non-parametric bootstrap procedure based on 1000 samples for all approaches and 500 samples for the parametric g-formula

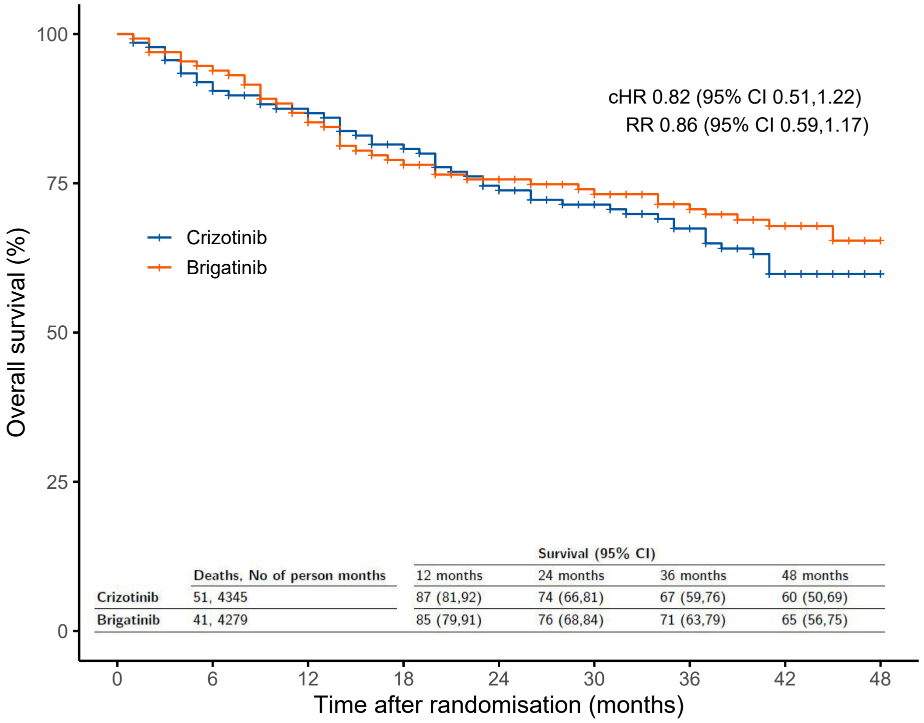

	Deaths, No of person months	Survival (95% CI) 12 months	24 months	36 months	48 months
Crizotinib	51, 4345	87 (81,92)	74 (66,81)	67 (59,76)	60 (50,69)
Brigatinib	41, 4279	85 (79,91)	76 (68,84)	71 (63,79)	65 (56,75)

(a) Kaplan-Meier survival curves comparing overall survival between "assign to brigatinib" and "assign to crizotinib" and assuming non-informative censoring; Survival probabilities are derived from the KM estimator, Cumulative Hazard Ratios (cHRs) and Risk Ratios(RRs) are estimated from a pooled logistic regression model

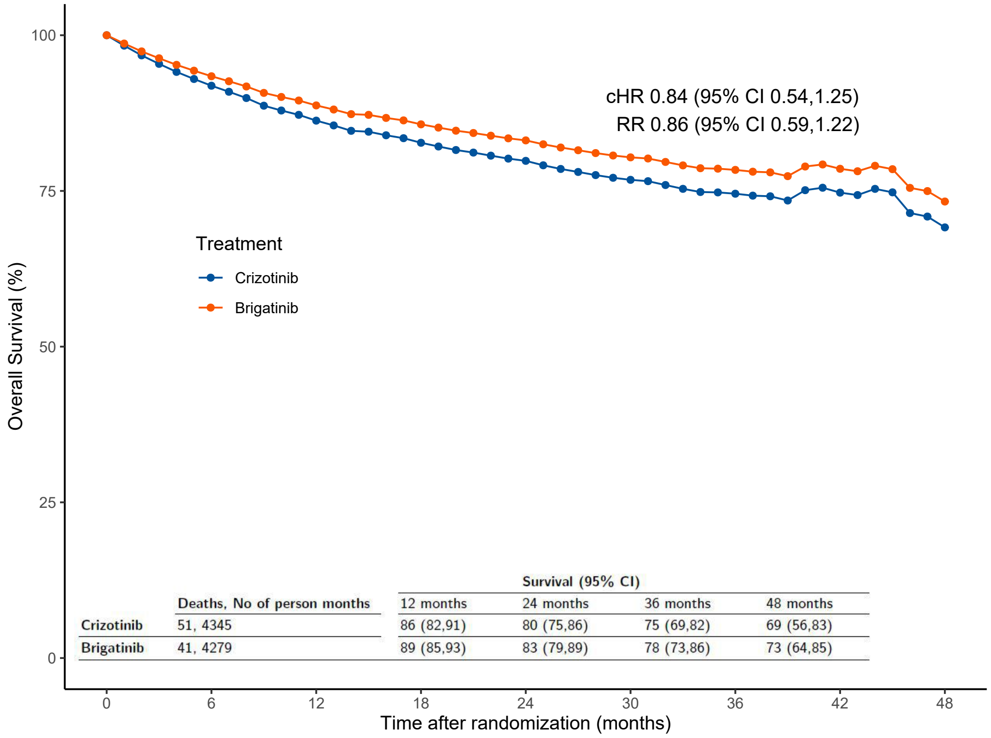

	Deaths, No of person months	Survival (95% CI) 12 months	24 months	36 months	48 months
Crizotinib	51, 4345	86 (82,91)	80 (75,86)	75 (69,82)	69 (56,83)
Brigatinib	41, 4279	89 (85,93)	83 (79,89)	78 (73,86)	73 (64,85)

(b) Parametric survival curves standardized for baseline covariate distribution from a pooled logistic regression comparing overall survival between "assign to brigatinib" and "assign to crizotinib" and assuming non-informative censoring; Cumulative Hazard Ratios (cHRs), Risk Ratios(RRs) and survival probabilities are estimated from a pooled logistic regression model standardized for baseline covariate distribution

Figure S2: Overall survival: ITT

Specification	RR	95% CI
1	0.87	0.61,1.20
2	0.86	0.60,1.17
3	0.87	0.61,1.18
4	0.87	0.61,1.19
5	0.85	0.59,1.15
6	0.88	0.62,1.20

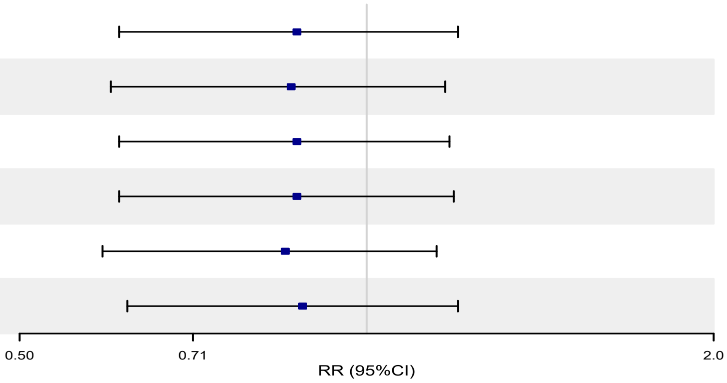

Figure S3: Forest plot of RR and 95% CI for the Causal effect of “assign to brigatinib” versus “assign to crizotinib” on OS investigated through specifications 1 to 6 using IPCW. Death is censored by LTFU/AC. RRs were estimated from a weighted pooled logistic regression using weights for LTFU/AC and formulated as the ratio of the cumulative risks by month 48. Reported CIs were estimated using a non-parametric bootstrap procedure based on 1000 samples. cHR, cumulative hazard ratio; CI, confidence interval; OS, overall survival, IPCW, inverse probability of censoring weights; LTFU, loss to follow up; AC, administrative censoring

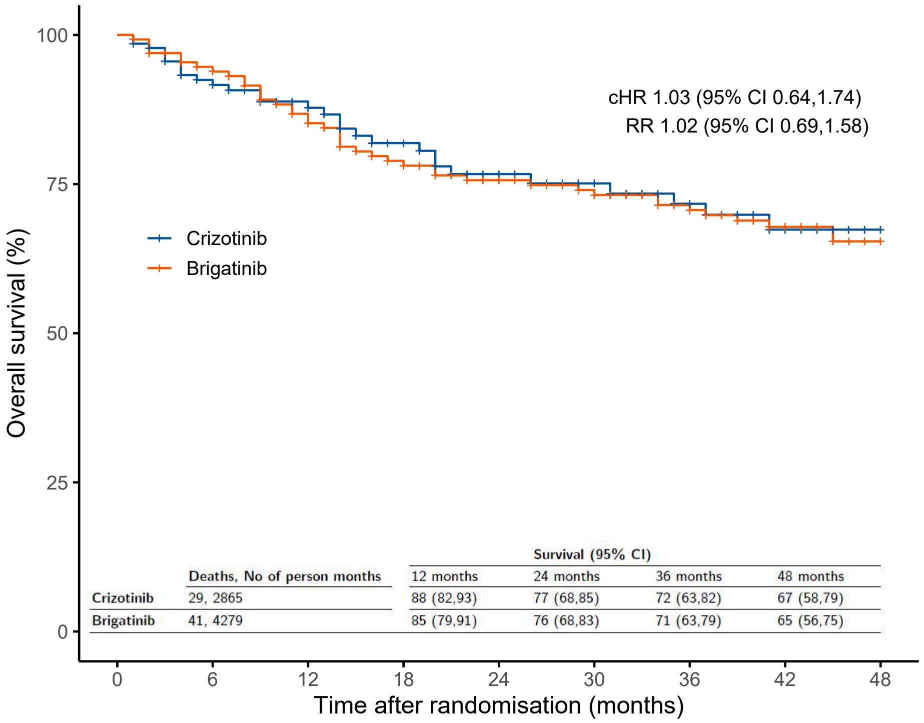

	Deaths, No of person months	Survival (95% CI) 12 months	24 months	36 months	48 months
Crizotinib	29, 2865	88 (82,93)	77 (68,85)	72 (63,82)	67 (58,79)
Brigatinib	41, 4279	85 (79,91)	76 (68,83)	71 (63,79)	65 (56,75)

(a) Overall survival: Censoring at switching

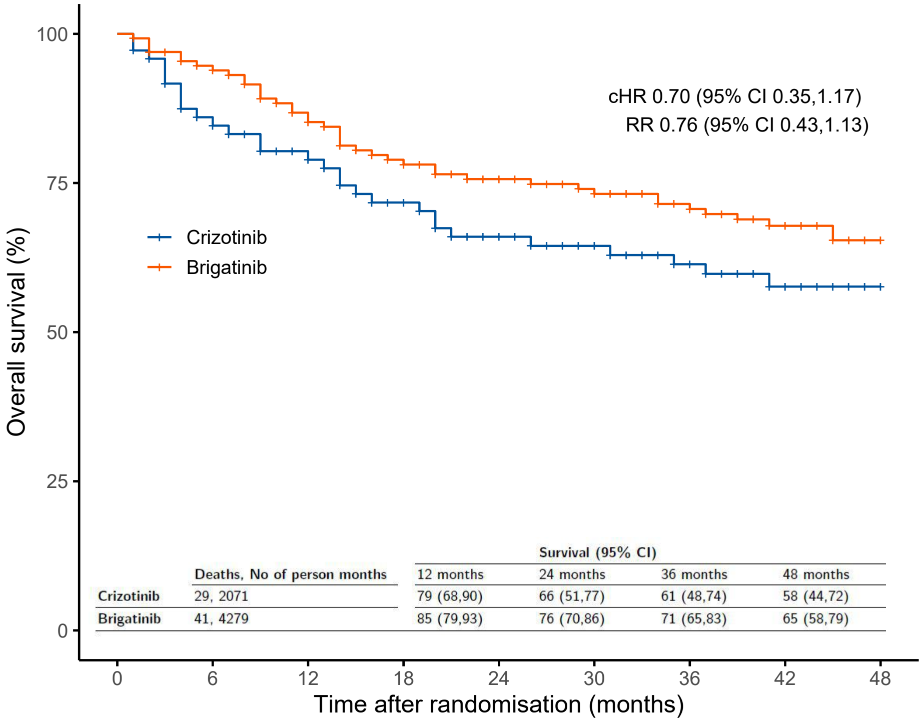

	Deaths, No of person months	Survival (95% CI) 12 months	24 months	36 months	48 months
Crizotinib	29, 2071	79 (68,90)	66 (51,77)	61 (48,74)	58 (44,72)
Brigatinib	41, 4279	85 (79,93)	76 (70,86)	71 (65,83)	65 (58,79)

(b) Overall survival: Excluding switchers

Figure S4: Kaplan-Meier survival curves comparing overall survival between "always Treat with Crizotinib" and "always Treat with brigatinib" and assuming non-informative censoring; Survival probabilities are derived from the KM estimator, Cumulative Hazard Ratios (cHRs) and Risk Ratios(RRs) are estimated from a pooled logistic regression model; In panel (a) switchers were excluded; In panel (b) switchers were censored at switching

Specification	RR	95% CI
1	0.75	0.49,1.16
2	0.75	0.51,1.16
3	0.74	0.49,1.20
4	0.75	0.49,1.16
5	0.72	0.43,1.17
6	0.79	0.54,1.17
7	0.53	0.32,1.16
8	0.52	0.32,0.98

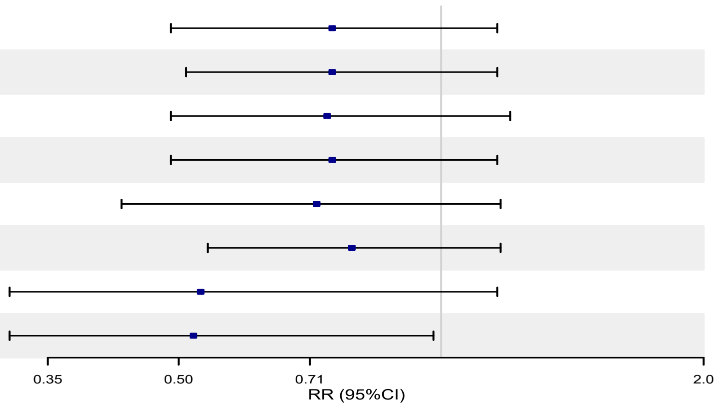

Figure S5: Forest plot of RR and 95% CI for the causal effect of “always treat with brigatinib” versus “always treat with crizotinib” on OS investigated through specifications 1 to 8 using IPCW. LTFU is assumed at random; Death is censored by a minimum of treatment switching and LTFU/AC and formulated as the ratio of the cumulative risks by month 48. Reported CIs were estimated using a non-parametric bootstrap procedure based on 1000 samples. cHR, cumulative hazard ratio; CI, confidence interval; OS, overall survival, IPCW, inverse probability of censoring weights

Specification	RR	95% CI
1	0.72	0.50,0.98
2	0.71	0.49,0.93
3	0.75	0.50,0.98
4	0.79	0.54,1.05
5	0.71	0.48,0.94

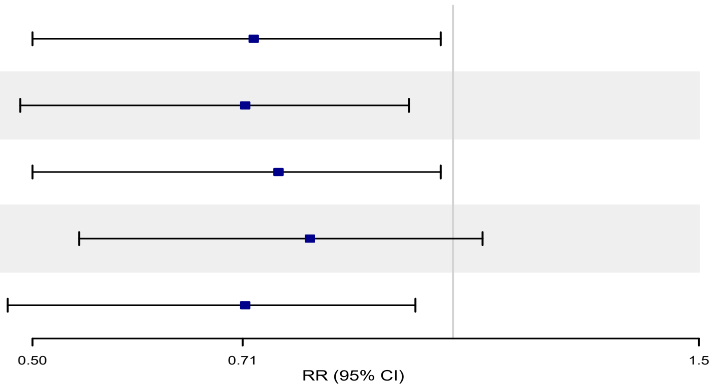

Figure S6: Forest plot of RR and their 95% CI for the causal effect of “always treat with brigatinib” versus “always treat with crizotinib” on OS investigated through specifications 1 to 5 using the parametric g-formula. Reported CIs were calculated using a non-parametric bootstrap procedure based on 500 samples (with 60 replicate failures observed for specification 4)
