# Supplementary material for: Addressing treatment switching in the ALTA-1L trial with g-methods: exploring the impact of model specification
Source: BMC Med Res Methodol. 2024 Dec 20;24:314. doi: 10.1186/s12874-024-02437-6 (PMC11660711; doi:10.1186/s12874-024-02437-6)
Supplement: Supplementary file 8 — Supplementary Material 8 provides results from the IPCW analyses using unstabilized weights. [file 12874_2024_2437_MOESM8_ESM.pdf]

Addressing Treatment Switching Bias with G-methods: Exploring the Impact of Model Specification

Amani Al Tawil*[1,2], Sean McGrath[3], Robin Ristl†[4], and Ulrich Mansmann†[1,2]

[1] *Institute for Medical Information Processing, Biometry, and Epidemiology (IBE), Faculty of Medicine, Ludwig-Maximilians-Universität München*
[2] *Pettenkofer School of Public Health, Faculty of Medicine, Ludwig-Maximilians-Universität München*
[3] *Department of Biostatistics, Harvard T.H. Chan School of Public Health*
[4] *Center for Medical Data Science, Medical University of Vienna*

Electronic Supplementary Material 8

IPCW analysis using unstabilized weights

*Correspondence: altawil@ibe.med.uni-muenchen.de
†Equally contributed

Table S10: Causal effect of "assign to brigatinib" versus "assign to crizotinib" on Overall survival (OS) investigated through various models (specification 1 to 6) for the construction of inverse probability of censoring weights for Lost to follow-up (LTFU)/administrative censoring (AC); Death censored by LTFU/AC only; 92 deaths, ALTA-1L trial

Specification	Description	Estimated unstabilized weights		Difference in OS	
		Mean (SD)	Min (Max)	cHR★ (95% CI)†	RR✪ (95% CI)†
1	Full model ✣	1.16 (1.32)	1 (94.02)	0.84 (0.52,1.28)	0.87 (0.59,1.22)
2	Restricted model ✣	1.17 (1.24)	1 (70.35)	0.82 (0.51,1.22)	0.85 (0.58,1.17)
3	Same as **specification 1**, but replace the linear terms of time, target lesion size and age with 5 knots splines	1.16 (1.10)	1 (56.93)	0.83 (0.51,1.29)	0.87 (0.59,1.21)
4	Same as **specification 1**, but replace the step function (3 categories) for time-varying ECOG with 2 categories	1.17 (1.58)	1 (123.81)	0.82 (0.50,1.26)	0.86 (0.58,1.20)
5	Same as **specification 2**, but replace the linear terms of time, target lesion size and age with 5 knots splines	1.17 (1.40)	1 (85.29)	0.80 (0.49,1.19)	0.84 (0.57,1.15)
6	Same as **specification 2**, but remove baseline covariates: sex, baseline ECOG and initial diagnosis stage in calculating the denominator of the experimental arm	1.18 (2.34)	1 (195.94)	0.84 (0.51,1.28)	0.87 (0.59,1.21)

✣ Details about the full and restricted model are available in Tables S1 and S2 in the Electronic Supplementary Material (ESM) 3)
★ cHR: Cumulative hazard ratio by month 48 (equation (5) in ESM 2).
✪ RR: Risk ratio formulated as the ratio of the cumulative risks by month 48.
† Reported 95% Confidence Interval (CI) estimated using a non-parametric bootstrap procedure based on 1000 samples

IPCW analysis using unstabilized weights

Table S11: Causal effect of "always treat with brigatinib" versus "always treat with crizotinib" on Overall Survival (OS) investigated through various models (specification 1 to 8) for the construction of the inverse probability of censoring weights for switching; Lost to follow-up (LTFU) assumed at random; Death censored by a minimum of treatment switching and LTFU/administrative censoring; 72 deaths, ALTA-1L trial

Specification	Description	Estimated unstabilized weights		Difference in OS	
		Mean (SD)	Min (Max)	cHR★ (95% CI)†	RR✪ (95% CI)†
1	Full Model ✜	1.13 (0.44)	1 (7.31)	0.68 (0.38,1.18)	0.74 (0.48,1.14)
2	Restricted Model ✜	1.14 (0.46)	1 (8.13)	0.68 (0.41,1.21)	0.74 (0.51,1.16)
3	Same as **specification 1**, but with 5 knots splines for time, time to disease progression, target lesion size and baseline age	1.13 (0.47)	1 (9.36)	0.65 (0.36,1.22)	0.72 (0.47,1.17)
4	Same as **specification 1**, but replace the step function (3 categories) for time-varying ECOG with 2 categories	1.13 (0.43)	1 (7.55)	0.68 (0.38,1.19)	0.74 (0.48,1.14)
5	Same as **specification 2**, but with 5 knots splines for time, time to disease progression, target lesion size and baseline age	1.14 (0.52)	1 (12.22)	0.62 (0.31,1.19)	0.70 (0.42,1.15)
6	Same as **specification 2**, but without step function (4 categories) for initial diagnosis stage	1.14 (0.46)	1 (4.82)	0.72 (0.44,1.22)	0.78 (0.53,1.17)
7	Same as **specification 1**, but without linear and quadratic terms for time to disease progression	1.37 (2.31)	1 (38.72)	0.40 (0.11,1.22)	0.52 (0.32,1.17)
8	Same as **specification 2**, but without linear and quadratic terms for time to disease progression	1.36 (2.03)	1 (36.72)	0.37 (0.12,0.99)	0.51 (0.32,0.99)

✜ Details about the full and restricted model are available in Tables S3 and S4 in the Electronic Supplementary Material (ESM) 3
★ cHR: Cumulative hazard ratio by month 48 (equation (5) in ESM 2).
✪ RR: Risk ratio formulated as the ratio of the cumulative risks by month 48.
† Reported 95% Confidence Interval (CI) estimated using a non-parametric bootstrap procedure based on 1000 samples
